# Process Optimization and Equilibrium, Thermodynamic, and Kinetic Modeling of Toxic Congo Red Dye Adsorption from Aqueous Solutions Using a Copper Ferrite Nanocomposite Adsorbent

**DOI:** 10.3390/molecules29020418

**Published:** 2024-01-15

**Authors:** Vairavel Parimelazhagan, Akhil Chinta, Gaurav Ganesh Shetty, Srinivasulu Maddasani, Wei-Lung Tseng, Jayashree Ethiraj, Ganeshraja Ayyakannu Sundaram, Alagarsamy Santhana Krishna Kumar

**Affiliations:** 1 Department of Chemical Engineering, Manipal Institute of Technology, Manipal Academy of Higher Education (MAHE), Manipal 576104, Karnataka State, India; pvairavel@gmail.com (V.P.); akhilchinta14@gmail.com (A.C.); shettygaurav00@gmail.com (G.G.S.); 2 Department of Chemistry, Manipal Institute of Technology, Manipal Academy of Higher Education (MAHE), Manipal 576104, Karnataka State, India; 3 Department of Chemistry, National Sun Yat-sen University, No. 70, Lienhai Road, Gushan District, Kaohsiung City 80424, Taiwan; tsengwl@mail.nsysu.edu.tw; 4 School of Pharmacy, Kaohsiung Medical University, No. 100, Shiquan 1st Road, Sanmin District, Kaohsiung City 80708, Taiwan; 5 Department of Physics, School of Arts and Science, AVIT Campus, Vinayaka Mission’s Research Foundation, Chennai 603104, Tamil Nadu State, India; dr.jayashreeethiraj@gmail.com; 6 CAS in Crystallography and Biophysics, University of Madras, Guindy Campus, Chennai 600025, Tamil Nadu State, India; 7 Department of Research Analytics, Saveetha Dental College and Hospitals, Saveetha Institute of Medical and Technical Sciences, Poonamallee High Road, Chennai 600077, Tamil Nadu State, India; 8 Faculty of Geology, Geophysics and Environmental Protection, Akademia Gorniczo-Hutnicza (AGH) University of Science and Technology, Al. Mickiewicza 30, 30-059 Krakow, Poland

**Keywords:** Congo red dye adsorption, copper ferrite nanocomposite, sol–gel synthesis, response surface methodology, adsorption kinetics, equilibrium isotherms, thermodynamics, reusability

## Abstract

In the present investigation of copper ferrite, a CuFe_2_O_4_ nanocomposite adsorbent was synthesized using the sol–gel method, and its relevance in the adsorptive elimination of the toxic Congo red (CR) aqueous phase was examined. A variety of structural methods were used to analyze the CuFe_2_O_4_ nanocomposite; the as-synthesized nanocomposite had agglomerated clusters with a porous, irregular, rough surface that could be seen using FE-SEM, and it also contained carbon (23.47%), oxygen (44.31%), copper (10.21%), and iron (22.01%) in its elemental composition by weight. Experiments were designed to achieve the most optimized system through the utilization of a central composite design (CCD). The highest uptake of CR dye at equilibrium occurred when the initial pH value was 5.5, the adsorbate concentration was 125 mg/L, and the adsorbent dosage was 3.5 g/L. Kinetic studies were conducted, and they showed that the adsorption process followed a pseudo-second-order (PSO) model (regression coefficient, R^2^ = 0.9998), suggesting a chemisorption mechanism, and the overall reaction rate was governed by both the film and pore diffusion of adsorbate molecules. The process through which dye molecules were taken up onto the particle surface revealed interactions involving electrostatic forces, hydrogen bonding, and pore filling. According to isotherm studies, the equilibrium data exhibited strong agreement with the Langmuir model (R^2^ = 0.9989), demonstrating a maximum monolayer adsorption capacity (q_max_) of 64.72 mg/g at pH 6 and 302 K. Considering the obtained negative ΔG and positive ΔH_ads_ and ΔS_ads_ values across all tested temperatures in the thermodynamic investigations, it was confirmed that the adsorption process was characterized as endothermic, spontaneous, and feasible, with an increased level of randomness. The CuFe_2_O_4_ adsorbent developed in this study is anticipated to find extensive application in effluent treatment, owing to its excellent reusability and remarkable capability to effectively remove CR in comparison to other adsorbents.

## 1. Introduction

The rapid expansion of modern industry has increased the emissions of potentially toxic synthetic dyes, posing grave global environmental threats to aquatic systems and other ecosystem habitats [1]. Groundwater and surface water pollution by inorganic and organic pollutants is a serious problem due to the high toxicity in the presence of dyes, and organic waste has become one of the world’s most severe issues [2]. Dyes are organic compounds with chromophores and auxochromes that adhere to a material’s surface and are used for coloring. They are utilized for dyeing purposes in a variety of industries, including the textile, paper, paint, food, pharmaceutical, cosmetic, and plastics industries [3]. One of the most water-intensive industries in India is the textile industry, which has more than 8000 units. The textile industry regularly generates colored effluents, which account for between 58 and 81% of the water used [4]. This is carried out to meet the continuous demand and to satisfy the needs of the growing population. The final washing and cleaning processes in the textile industries require a great deal of water, in addition to the printing and dying processes (40 L of water is used in the dyeing process for every kg of fabric dyed) [5]. The synthetic dye Congo red (CR) is highly dissolvable in water and stable in air and light, with a complex aromatic structure; the molecular formula of CR is C_32_H_22_N_6_Na_2_O_6_S_2_. It is an anionic acid dye used in laboratories as a histological stain for amyloid, in tests of free HCl in gastric content, and as a diagnostic tool for amyloidosis [6]. The presence of one or more –N=N– groups bound to aromatic rings like benzene and naphthalene is a characteristic feature of the structure of azo dyes [7]. Azo dyes consist of aniline compounds, which are hazardous substances capable of causing significant environmental pollution and posing a threat to the health of both humans and animals due to their toxic nature [8]. In wastewater with different pH values, CR exists in different molecular forms, which makes it difficult to degrade due to the presence of azo groups [9]. Even a low concentration of CR dye can cause several negative effects, including difficulty breathing; blood clotting; harm to the kidneys, liver, and respiratory system; allergic dermatitis; skin, eye, and gastrointestinal irritation; diarrhea; nausea; vomiting; pain in the abdomen and chest; and severe headache. CR dye is a carcinogen, mutagen, and reproductive effector; is an important source of water pollution; and is extremely challenging to remove because of its intricate structure [10]. Therefore, it is imperative to promptly eliminate Congo Red (CR) from industrial wastewater to safeguard both the aquatic ecosystem and human life. This action should be taken before the release of dye effluents into the environment [11].

Different techniques, such as chemical precipitation, membrane separation, adsorption, ion exchange, photo-catalytic degradation, electrochemical oxidation, ozonation, the Fenton process, and sonication, have been extensively employed for the remediation of wastewater containing dye contaminants [12]. However, these methods require huge investments, and the operating costs are also high. Adsorption is a well-known separation process due to its initial cost, rapid technique, simplicity of design, ease of operation, insensitivity to toxic pollutants, environmental benignity, and the regeneration and reusability of adsorbents, and because it does not produce any harmful substances [13]. This method is proven to be cost-effective and features a notable adsorption rate. It is also suitable for effluent treatment plants with minimal infrastructure and equipment requirements and does not require expensive chemicals or a source of clean water [14,15]. Activated carbon has been commonly used as an adsorbent to remove toxic pollutants from effluents. However, its high cost limits its use on a large scale, and it is not easy to regenerate [16]. Nanostructured materials with unique physicochemical properties offer a promising solution for efficient adsorption. Ideally, such materials should be environmentally friendly, have a high adsorption capacity, be selective and reusable, and facilitate the easy removal of the adsorbate [17]. Mixed metal oxide nanocomposites are currently of great interest to researchers due to their broad range of catalytic, electronic, and magnetic properties and their suitability for heterogeneous catalysis [18]. These novel nanocomposites have a large surface area and pore volume, making them highly effective for the adsorption and removal of toxic pollutants from wastewater. Nanocomposites can effectively adsorb and capture dye molecules, making them an ideal choice for effluent treatment systems. These composites can be customized to meet specific requirements by controlling their pore size and chemical composition. This targeted approach allows for the selective attraction and retention of dye molecules, making them highly efficient for pollutant removal. Additionally, nanocomposites are cost-effective and require less energy than conventional processes, making them a practical choice for wastewater purification [19]. Many studies have reported the use of metal oxide nanocomposites such as Fe_2_O_3_-Al_2_O_3_, Fe_3_O_4_-Graphene oxide, Ni-Fe_2_O_4_, Fe_2_O_3_-MnO_2_-SnO_2_, MgO-MgFe_2_O_4_, ZnTiO_3_-Zn_2_Ti_3_O_8_-ZnO, and SnO_2_-Fe_3_O_4_ for the treatment of wastewater containing toxic substances [20]. Copper (Cu) and iron (Fe) oxides are notable among the array of metal oxides due to their advantages, such as low costs, widespread availability, thermal stability, and high adsorption capacity. In addition, copper-based nanocomposite materials can be prepared easily in multiple ways [21]. Pooja and Mala investigated mixed metal oxide nanocomposites prepared by the sol–gel method used as adsorbents to remove toxic organic pollutants from aqueous systems [22]. Beyki et al. studied a copper ferrite–polymer nanocomposite prepared using the chemical precipitation method to remove methylene blue from simulated effluents [23]. Xie et al. synthesized mesoporous Mg–Al mixed metal oxide nanocomposites and studied their adsorption capacity for CR dye [24]. Deflaoui et al. synthesized graphene oxide–TiO_2_ nanocomposites as an adsorbent to remove methyl orange dye [25]. Sharma et al. examined the synthesis and use of a CuO–ZnO tetrapodal hybrid nanocomposite as an adsorbent to remove an anionic dye, reactive yellow, and a cationic dye, basic violet [26]. Liu et al. described the synthesis of a magnetic Fe_3_O_4_–graphene oxide composite and its use in the dye removal of methylene blue from aqueous media [27]. However, the problem with adsorbent regeneration has not yet been resolved. Thus, developing materials with strong adsorption and regeneration abilities is crucial in the adsorption process. Spinels are a group of compounds denoted as M^2+^M_2_^3+^O_4_, garnering significant research interest due to their multifaceted properties and widespread applications across diverse domains. By setting M^3+^ as Fe, we derive spinel ferrites following the general formula MFe_2_O_4_, while transitioning metal spinel ferrites arise when M encompasses elements like Cu, Fe, Mn, Ni, and Zn, among others, positioned at tetrahedral and octahedral sites. These spinel ferrites have garnered increased attention for their magnetic and semiconducting characteristics [21]. Among the various ferrites, spinel copper ferrite holds significant promise, serving as a ferrofluid, a humidity and gas sensor, and a catalyst in organic reactions, and playing roles in applications like multilayer chip induction, high-speed digital tapes and recording disks, and rod antennas [28]. However, studies have not been conducted on the removal of CR from the aqueous phase using a copper ferrite (CuFe_2_O_4_) nanocomposite adsorbent. To our knowledge, there has been no previous investigation into the application of copper ferrite as an adsorbent, whether in batch or continuous mode, to eliminate aqueous solutions containing CR dye. Therefore, it is essential to conduct studies evaluating the effectiveness of copper ferrite as an affordable nanocomposite for the elimination of color from contaminated water. The use of specific copper-based ferrite nanocomposites as a new adsorption technology for the removal of CR, an anionic dye from synthetic effluents, is an interesting and potentially effective approach. The newly developed copper ferrite nanocomposite has been tested in simulated wastewater samples, and its efficacy in removing these dye molecules has been demonstrated. The use of this nanocomposite reduces the concentration of this detrimental pollutant in the effluent to levels deemed safe for discharge into the environment [21].

The optimization of the experimental factors for CR dye removal with the CuFe_2_O_4_ nanocomposite adsorbent has not been previously conducted using response surface methodology (RSM) investigations. The RSM model has been extensively applied in various scientific research domains [29]. In the realm of adsorbent materials, the RSM model is employed to assess the optimal adsorption capacity, allowing for the optimization of the experimental conditions for nanocomposite materials. This research demonstrates an easy preparation method (sol–gel) for a novel mixed metal oxide nanocomposite for the effective adsorption of the toxic CR dye from the aquatic phase. Experiments were conducted with varying process parameters, such as the initial pH, nanocomposite adsorbent dosage, size, and adsorbate concentration, to study their effect on the adsorption process. We can confidently and effectively optimize the process parameters to guarantee the maximum equilibrium dye uptake by implementing a central composite response surface design. The properties of the prepared nanocomposite material were studied using various characterization techniques to investigate its reusability in multiple runs. Furthermore, to design effective adsorption systems, it is crucial to examine the adsorptive capacity of the optimal value of the nanocomposite under batch conditions and understand the adsorption process mechanism. Thus, the CR adsorption isotherm, kinetics, and thermodynamics were considered to explore its adsorptive capacity and gain insights into the adsorption process.

## 2. Results and Discussion

### 2.1. Characterization of the Copper Ferrite (CuFe_2_O_4_) Nanocomposite Adsorbent

**Figure 1:**
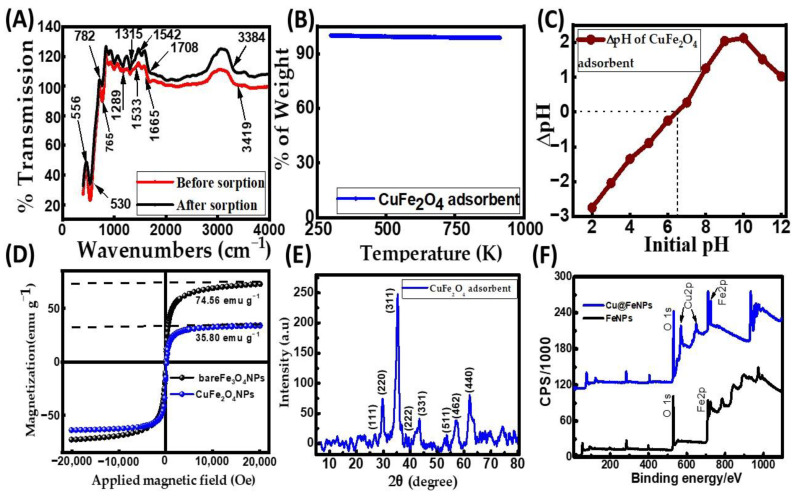
Characterization of the CuFe_2_O_4_ nanocomposite adsorbent: (**A**) Fourier transform infrared spectroscopy (FT-IR) spectra; (**B**) thermogravimetric analysis (TGA) profile; (**C**) zero-point charge (pH_zpc_) graph; (**D**) magnetic hysteresis profile; (**E**) X-ray diffraction (XRD) analysis; and (**F**) X-ray photoelectron spectroscopy (XPS) spectra.

The Fourier transform infrared spectroscopy (FT-IR) spectrum of the synthesized CuFe_2_O_4_ nanocomposite adsorbent before and after CR dye adsorption is presented in Figure 1A, with distinctive absorption in the low-frequency region. The spectra give information about the chemical species and molecular structure, indicating the functional groups in the synthesized ferrite. Different absorption peak locations of the tetrahedral and octahedral sites of the copper ferrite nanocomposites have been observed due to the variations in the bond length values of the Fe (Cu)–O bond [30]. Patil et al. discussed the movement behavior of spinel ferrite, describing the rearrangement of phase transformation and cations [31]. The FT-IR spectrum of the adsorbent to CR dye adsorption reveals a distinct and intense peak at 3419.63 cm^−1^, indicative of the stretching vibrations of the –OH groups associated with adsorbed moisture [32]. The CuFe_2_O_4_ nanocomposite peaked at 1533.85 cm^−1^, attributed to the O–H bending vibration of H_2_O molecules present in the compound. A similar–OH stretching vibration in the copper ferrite nanocomposite has been observed [30,33]. The distinct peak at 1289.62 cm^−1^ is ascribed to the stretching vibration of the metal oxide within the hexagonal sites of the crystalline structure. The slender and sharp peak observed at 530.44 cm^−1^ is attributed to the octahedral group Cu–O stretching vibration, signifying the crystal structure’s absorption properties [34]. The absorption band at 765.38 cm^−1^ is due to the tetrahedral group Fe–O stretching bands. After dye adsorption, a shift was observed. The peaks of hexagonal sites’ metal–oxide stretching vibration, Fe–O bond stretching vibration, and the stretching vibration of Cu–O shifted from 1289.62 cm^−1^, 530.44 cm^−1^, and 765.38 cm^−1^ to 1315.22 cm^−1^, 556.08 cm^−1^, and 782.54 cm^−1^, respectively. This result indicates that the CR dye molecules were adsorbed on the surface of the nanocomposite adsorbent. These metal–oxide bonds and metal ions on the adsorbent surface could potentially serve as binding sites for electrostatic interactions with the negatively charged adsorbate molecules, thereby promoting the decolorization of the dye solution. The thermogravimetric analysis (TGA) result of the nanocomposite adsorbent is shown in Figure 1B. It is clear from the TGA curve that the nanocomposite adsorbent remains almost entirely stable, with little to no weight loss even after being heated up to 1073 K. This result reflects that the adsorbent is highly pure and thermally stable at high temperatures. In other words, the particle surface has no moisture or volatile matter [21]. From the Brunauer–Emmett–Teller (BET) measurement, the nanocomposite adsorbent’s pore volume and surface area were determined to be 71 mm^3^/g and 30.03 m^2^/g, respectively. The adsorbent had an average particle size of 742.5 nm. The solid particle surface’s pH at the point of zero charge (pH_zpc_) was ascertained using the salt addition method [35], and it was 6.5 using the data shown in Figure 1C. It was observed that the particle surface of the CuFe_2_O_4_ nanocomposite became positively charged at pH levels below 6.5, and it would be negatively charged at a pH greater than 6.5. A superconducting quantum interference device (SQUID) magnetometer was employed to study the magnetic properties of CuFe_2_O_4_ nanoparticles (CuFe_2_O_4_ NPs) and Fe_3_O_4_ nanoparticles (Fe_3_O_4_ NPs) at room temperature (Figure 1D). Compared with bare Fe_3_O_4_ NPs (74.56 emu g^−1^), the decreased magnetization saturation value of CuFe_2_O_4_ NPs (35.80 emu g^−1^) resulted from the less magnetic behaviors of CuFe_2_O_4_ NPs. Moreover, similar to the magnetic properties of Fe_3_O_4_ NPs, CuFe_2_O_4_ NPs exhibited remanence-free and zero coercivity magnetization curves with S-shaped hysteresis lines in response to an applied magnetic field at room temperature [36]. These observations reflect that CuFe_2_O_4_ NPs possess super-paramagnetic behavior.

**Figure 2:**
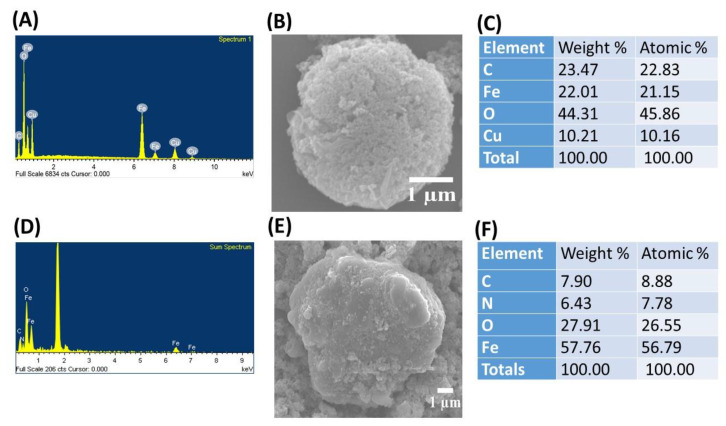
Energy-dispersive X-ray spectroscopy (EDS)/field-emission scanning electron microscopy (FE-SEM) images of (**A**–**C**) copper ferrite (CuFe_2_O_4_) nanocomposite adsorbent; and (**D**–**F**) bare Fe_3_O_4_ NPs.

The X-ray diffraction (XRD) pattern shown in Figure 1E was analyzed for the crystallographic identity of the CuFe_2_O_4_ nanocomposite. The Cu^2+^ ions inhabit the tetrahedral sites in the crystal lattice, while Fe^3+^ ions occupy the octahedral sites. This arrangement is supported by the diffraction peaks and their corresponding Miller indices in the XRD pattern, indicating that the prepared CuFe_2_O_4_ possesses a cubic spinel structure. The presence of narrow and sharp diffraction peaks confirms the existence of a well-developed crystalline structure, consistent with the standard JCPDS file No: 00-077-0010. It was confirmed that the required copper ferrite nanocomposite was achieved. Additionally, traces of copper oxide are associated with the JCPDS card No. 48-1548 [28]. The diffraction peaks in Figure 1E correspond to the (h k l) planes (111), (220), (311), (222), (331), (511), (422), and (440), respectively, at angles of 2ϴ (°) = 27, 30, 35, 38, 42, 54, 58, and 63 degrees, indicating a high degree of crystallinity. Scherrer’s equation [37], as provided in Equation (1), was used to compute the crystallite size (D) using the line broadening of the peak with the highest intensity (311) (full width at half-maximum (FWHM), β as a base):

(1) $$D\;=\frac{0.9\times \;\lambda }{\beta \;\cos\;\theta }$$

where θ represents the Bragg angle, and λ is the X-ray wavelength (1.54 Å). The average crystallite size of the CuFe_2_O_4_ nanocomposite was determined to be 18.64 nm. The chemical composition of the CuFe_2_O_4_ nanoparticles (NPs) was analyzed using X-ray photoelectron spectroscopy (XPS). The total survey XPS spectra indicated the presence of Fe, O, and Cu elements in the as-prepared adsorbent of CuFe_2_O_4_ NPs, as shown in Figure 1F. This observation was further confirmed by field-emission scanning electron microscopy (FE-SEM) coupled with energy-dispersive X-ray spectroscopy (EDS), demonstrating that the prepared nanoparticle (NP) was composed of Fe, O, Cu, and C elements (Figure 2A). Iron arises from Fe_2_O_4_ NPs, while Cu arises precursors from cupric nitrate trihydrate (Cu(NO_3_)_2_·3H_2_O). This feature suggests that the as-prepared nanoparticle is well suited for organic dye elimination. The surface morphology of the prepared adsorbent was characterized using FE-SEM. Figure 2B shows the micrograph image of copper ferrite at 10 kX magnification before CR dye adsorption. Figure 2B illustrates that the rough surface of the copper ferrite adsorbent is irregular with agglomerated clusters. This design creates significant voids and pores, allowing adsorbate molecules to accumulate on the nanocomposite particle surface. The nanocomposite agglomeration is the typical thermal dissociation product. The exothermic process’ gaseous by-product (NO_3_) is responsible for the voids and porous structure. A similar surface morphology image has been shown in the literature [28]. The EDS analysis of the CuFe_2_O_4_ nanocomposite adsorbent before CR dye removal is shown in Figure 2A. The sharp peaks observed in the EDS spectrum (Figure 2A) validate the existence of Cu (1 keV, 8 keV, and 8.9 keV), Fe (0.6 keV, 6.4 keV, and 7 keV), and oxygen (0.5 keV), exhibiting similarity to the findings reported in a published article [38]. The elemental composition of the nanocomposite was identified through analytical FE-SEM with EDS at 20 keV. The formation of the CuFe_2_O_4_ nanocomposite became evident, with weight proportions of copper, Fe, oxygen, and carbon at 10.21%, 22.01%, 44.31%, and 23.47%, respectively (Figure 2C). The confirmation of CuFe_2_O_4_ nanocomposite formation and the capping by molecules was established through the detection of carbon [39]. After the adsorption process, there was an observed increase in both the weight and atomic percentages of elemental carbon and oxygen. This implies that the surface of the nanocomposite material became laden with anions of the CR dye.

### 2.2. Effect of Initial pH of Dye Solution on Adsorption Behavior

The initial pH of the dye solution is the essential factor that affects the adsorption capacity. Figure S2 displays the structure of the CR dye. Studying the effect of the initial pH on the uptake of acidic CR dye is quite difficult as it can result in the formation of protonated species, which may alter the structure of the dye. At acidic pH levels (pH < 5.5), the CR dye appears black due to the creation of a quinonoid complex structure in the aqueous phase [40]. The pH of the CR dye remained consistent within the range of 5.5 to 12; however, it became unstable when the solution’s pH dropped below 5.5. The impact of the initial pH on CR dye uptake was investigated by adjusting the initial pH of the dye solution from 5.5 to 12, and the outcomes are depicted in Figure S3A. It shows that the adsorption capacity of CR diminished from 45.89 to 4.17 mg/g with a rise in solution pH from 5.5 to 12. This could be attributed to the escalation of electrostatic repulsive forces among the CR dye molecules and the negatively charged surface of the CuFe_2_O_4_ nanocomposite adsorbent when the pH exceeds 6.5. As a result of this electrostatic repulsion, a negatively charged surface site on the particle surface does not support the adsorption of CR dye anions. It was found that the optimum pH level for the solution was 5.5. The quantity of negatively charged sites increases with the elevation of the solution’s pH, as an augmented abundance of surplus OH^−^ ions deprotonates the binding sites on the adsorbent’s surface. Between the protonated binding sites of the adsorbent and anionic dye molecules at pH 5.5, there is strong electrostatic attraction and van der Waals forces. Additionally, there is competition for adsorption binding sites between the surplus OH^−^ ions and the negatively charged dye molecules, leading to the reduced adsorption of CR at basic pH levels [41].

### 2.3. Experimental Design for Process Parameter Optimization

A statistical design of experiments was employed through the response surface methodology (RSM) to streamline the optimization process and minimize the number of experimental trials. This approach investigated the relationship between process factors and the equilibrium dye uptake. The statistical program Minitab 16 was used to create the experimental design, and the central composite design (CCD) was used to carry out the adsorption experiments. The CCD establishes the regression model and investigates how different process factors interact in the system. The experiment considered the independent variables of the initial pH of the dye solution (X_1_), the initial concentration of the adsorbate (X_2_), and the CuFe_2_O_4_ nanocomposite adsorbent dosage (X_3_) as influencing factors, while the response variable was the equilibrium dye uptake. The experiments were designed with three levels (−1, 0, and +1) to investigate the impacts of these variables. The calculation of the number of experimental runs (N) was determined using the following Equation (2) [42]:

N = 2^k^ + 2k + N_o_ (2)

where k is the number of independent variables, 2k is the number of factorial points, and 2k is the number of axial points; N_o_ denotes the center points. A total of 20 experimental trials were performed, utilizing a 23 complete factorial design involving 8 cube points, 6 axial points, and 6 center points. The process variables’ coded values were taken from Equation (3) [37]:

(3) $$x_{i}=\frac{\left(X_{i}-X_{o}\right)}{\delta X}\;i\;=\;1,\;2,\;3,\;\ldots ,n$$

where x_i_ is a process variable’s dimensionless value; X_i_ represents an independent variable’s actual value. At the center point, X_i_ equals X_o_, and δX denotes the step change. Table 1 lists the experimental ranges and values of different process parameters that remove CR dye.

The relationship among the independent and the process response variables is expressed as a second-order polynomial expression stated as [43]

(4) $$Y_{p}={\;\alpha }_{o}+\overset{n}{\underset{i=1}{\sum }}\left(\alpha_{i}X_{i}\right)+\overset{n}{\underset{i=1}{\sum }}\left(\alpha_{\mathrm{ii}}X_{i}^{2}\right)+\overset{n}{\underset{i,j=1,\;i\neq j}{\sum }}\left(\alpha_{\mathrm{ij}}X_{i}X_{j}\right)$$

Y_p_ represents the estimated response variable from the RSM in the given context. α_o_ is the coefficient offset term, and α_i_, α_ii_, and α_ij_ are the regression coefficients for the linear, quadratic, and interaction impacts, respectively. The sign of each coefficient indicates how the relationship correlates with the response variable. To validate the predicted data from RSM, the expected responses are compared to the experimental findings. The model’s precision is assessed using the root mean square error (RMSE) and the absolute average deviation (AAD), calculated by the following equations [44]:

(5) $$\mathrm{RMSE}=\sqrt{\left(\frac{1}{N\;}\;\sum {\left(Y_{a}-{\;Y}_{p}\right)}^{2}\right)}$$

(6) $$\mathrm{AAD}=\frac{1}{N}\;\sum \left(\frac{{\;Y}_{p}-{\;Y}_{a}}{Y_{a}}\right)\times 100$$

where Y_a_ refers to the experimental output value, and Y_p_ denotes the calculated output value acquired from the RSM.

#### 2.3.1. Analysis of Experimental Design and Process Parameter Optimization

Using statistically constructed experiments, several independent variables are utilized to examine the interactions between various parameters. Table 2 displays the results of comparing the calculated output values with 20 sets of experimental data.

According to the data in Table 2, experiment number 12 achieved the highest adsorption capacity at equilibrium, reaching 48.72 mg/g. Table 3 displays the outcomes of the analysis of variance (ANOVA) conducted on the results. From Table 3, the most significant factors in the analysis are the linear effects of the initial dye concentration (X_2_), CuFe_2_O_4_ nanocomposite adsorbent dosage (X_3_), and initial pH of the dye solution (X_1_), which have coefficients with *p*-values of 0.000, 0.000, and 0.027, respectively [44]. The X_2_X_3_ interaction effect coefficient, which has a positive impact, is the most crucial factor, with a significance level of *p* = 0.003. However, the coefficients of the quadratic effect between various independent variables and the other interactive effects (X_1_X_2_ and X_1_X_3_) do not appear to be statistically significant. The regression model (Equation (7)) for the equilibrium dye uptake is

CR dye uptake at equilibrium = 40.6965 − 0.5309 X_1_ − 2.7446 X_2_ − 3.9479 X_3_ − 0.0594 X_1_^2^ − 0.7069 X_2_^2^ − 0.0416 X_3_^2^ + 0.4239 X_1_X_2_ + 0.4701 X_1_X_3_ + 0.9099 X_2_X_3_ (7)

The high regression coefficient, or R^2^ value (98.34%), indicates that the calculated values produced from the model are close to the empirical values. The anticipated R^2^ (87.04%) can be determined using the predicted residual sum of squares (PRESS) statistics. This measure helps to prevent the model from overfitting. A tool used to assess the fit quality is the adjusted R^2^ (96.81%), but it is more suitable for contrasting the model with different independent variables. By utilizing degrees of freedom in its computations, the R^2^ value is adjusted for both the sample size and the number of terms in the model. The equation for the best-fit model has a lower RMSE (0.4587) and AAD (0.84%) value. Hence, the influence of noise on the quadratic model is minimal. This is validated by the higher adequate precision value (17.643), which signifies a favorable signal-to-noise ratio. Besides the ANOVA findings, diagnostic plots were acquired to validate the chosen quadratic model. The residual is the difference between the projected and actual values, providing a measure to evaluate the quadratic model’s alignment with the data. Hence, the analysis of residuals plays a vital role in confirming the appropriateness and significance of the derived quadratic model in accurately predicting the response. The residual error, assessing the disparity between the experimental and calculated output values, is employed to evaluate the model’s adequacy and conformity to a normal distribution [42].

**Figure 3:**
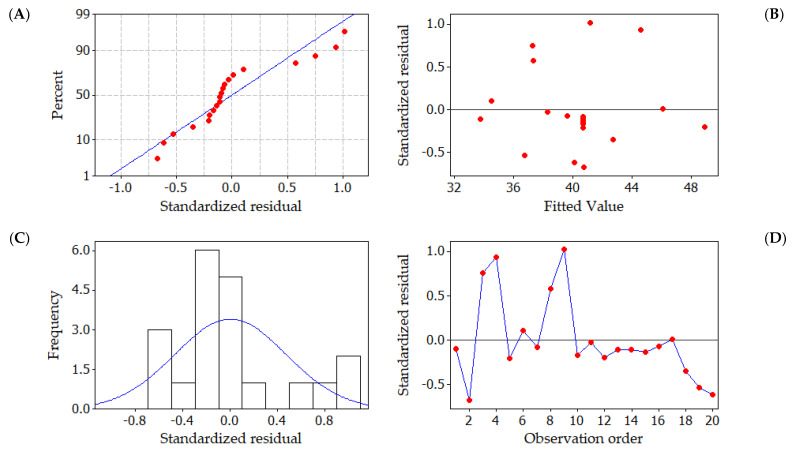
Graphical representations of residuals in CR dye uptake onto CuFe_2_O_4_ adsorbent. (**A**) Standard probability plot of standardized residuals, Red dot indicates that the residuals in the plot follow a straight line (**B**) fitted values plotted against standardized residuals, (**C**) frequency of observation versus standardized residuals, Blue line indicates that the normal distribution of the standardized residuals and (**D**) standardized residuals plotted against data order, Red dot indicate the standardized residuals in the order of corresponding observations. Blue line indicates that the residuals in the plot fluctuate in a random pattern.

Figure 3A depicts a graph illustrating the relationship between the normal percentage probability and residuals. In Figure 3A, it is evident that all residual values adhere to a typical distribution pattern. Furthermore, the residuals in the plot are almost entirely aligned with the diagonal line, indicating close correspondence between the chosen quadratic model and the actual data. The analysis residuals are the best residuals, but they do not have any impact on the outcome. Figure 3B depicts the standardized residual versus fitted values plot. In this plot, the residuals seem to be randomly dispersed above and below the zero lines. The broader distribution of residuals in this plot indicates the increased fitted values. The histogram of standardized residuals is displayed in Figure 3C. The plot’s extended tail shows a skewed distribution. Outliers could be the one or two bars that stand out. The better-fitting values are represented by the non-uniform bars in the plot. The standardized residuals are shown in Figure 3D; in the corresponding run number, the residuals are observed not to follow a particular trend, suggesting that the data variance is almost constant and the distribution is irregular. The non-random error was calculated using the observation that the residuals in the plot move erratically above and below the zero lines in the observation sequence [45]. The analysis of residuals leads to the inference that the formulated quadratic model to enhance the uptake efficiency of CR dye onto the CuFe_2_O_4_ nanocomposite is both precise and well suited for the prediction of the response.

#### 2.3.2. Analysis of Contour and Response Surface Plots

**Figure 4:**
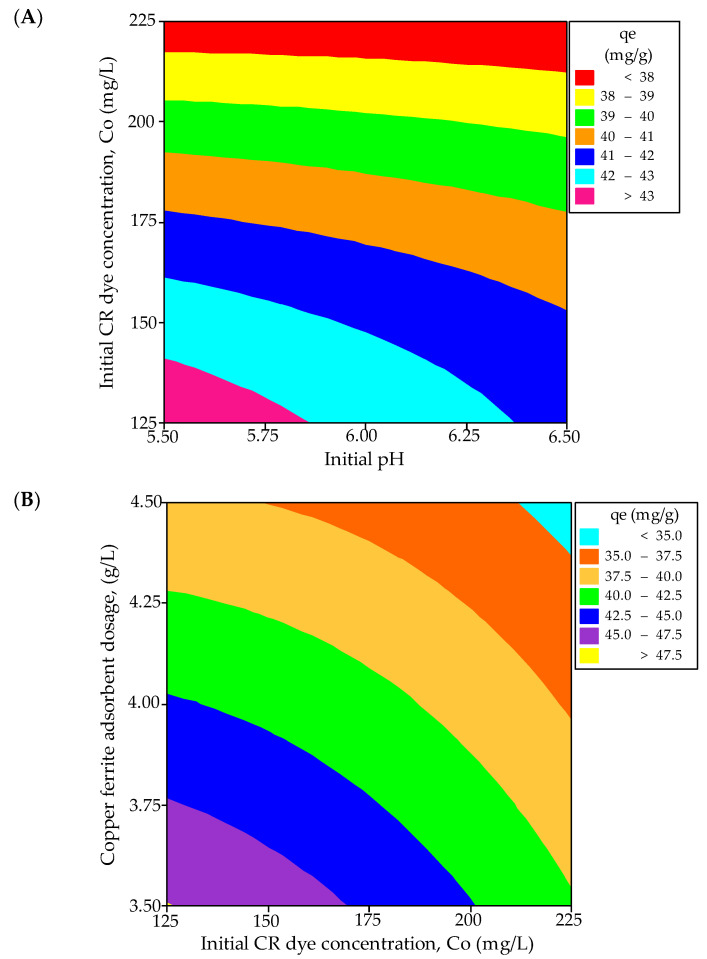
Contour plots for the interactive effect of (**A**) initial dye concentration and pH, and (**B**) CuFe_2_O_4_ adsorbent dosage and initial adsorbate concentration, on the equilibrium dye uptake of CR.

**Figure 5:**
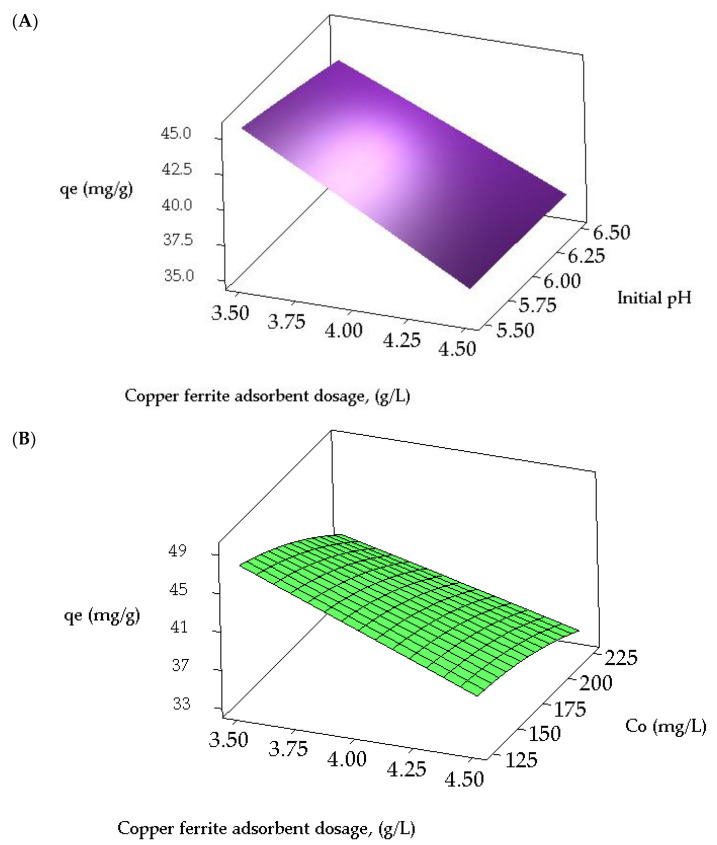
Response surface plots for the interactive effect of (**A**) initial pH and CuFe_2_O_4_ adsorbent dosage, and (**B**) initial adsorbate concentration and dosage of CuFe_2_O_4_ adsorbent, on the equilibrium uptake of CR dye.

Contour and three-dimensional (3D) response surface plots are employed to investigate the interrelationships among the variables and ascertain the peak response levels for each variable. Figure 4A,B illustrate the contour plots representing the adsorption capacity of CR from a synthetic effluent. Figure 4A presents a contour plot depicting the equilibrium CR dye uptake concerning the initial adsorbate concentration and pH. The optimal dye uptake is observed within the 125 to 138 mg/L range for the adsorbate concentration and 5.5 to 5.875 for the initial pH. Additionally, the interaction effect between these variables is found to be significant. Figure 4B illustrates that the highest predicted yield is observed when the CuFe_2_O_4_ adsorbent dosage falls within the range of 3.5 to 3.51 g/L and the initial adsorbate concentration varies between 125 to 126 mg/L, with a significant impact. The 3D surface plots illustrating the equilibrium dye uptake of CR from simulated dye wastewater are shown in Figure 5A,B. Figure 5A indicates a decrease in the dye adsorption capacity at equilibrium with higher pH values and an increased CuFe_2_O_4_ adsorbent dosage. The response plot, covering the initial pH of the adsorbate solution from 5.5 to 6.5 and a particle dosage ranging between 3.5 and 4.5 g/L, clearly demonstrates a significant impact on the equilibrium dye uptake. Likewise, Figure 5B illustrates an enhancement in adsorption capacity with a decrease in adsorbent dosage and an increase in the initial adsorbate concentration. The response surface plot, encompassing dye concentrations ranging from 125 to 225 mg/L and adsorbate concentrations between 3.5 and 4.5 g/L, significantly influences CR uptake from the liquid phase. The maximum response values obtained from the response surface plots nearly align with those acquired from the experimental data and the regression model equation.

#### 2.3.3. Reliability of the Process Model

After validating the developed quadratic model by ANOVA and residual analysis, the model was used to predict the response variable, i.e., equilibrium adsorption capacity. Three solutions, each having distinct values of ideal conditions, were employed to predict the optimal conditions for CR dye uptake at equilibrium using the CuFe_2_O_4_ nanocomposite adsorbent, as presented in Table 4. Different experiments were run with varying degrees of the experimental parameters, and the outcomes were compared to the predictable responses. According to Table 4, experiment number 2 achieved the highest adsorption capacity of 48.72 mg/g compared to the other two experiments. The predicted value of 49.38 mg/g, obtained using the regression model, was favorably aligned with the experimental value of 48.72 mg/g. The close agreement between the experimental responses and their calculated values indicates that the model derived from the design effectively explains the positive relationship between the various independent variables and the CR dye uptake at equilibrium [46]. Table 5 presents the optimal values of the process-independent variables that lead to the maximal response. The optimization studies unambiguously demonstrate that RSM is a suitable strategy to maximize the optimal operational parameters to achieve the maximum equilibrium dye uptake.

### 2.4. Isotherm Modeling

**Figure 6:**
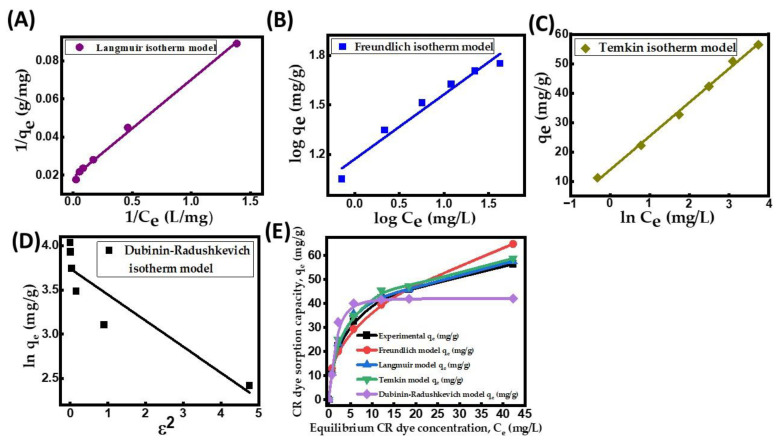
CR dye uptake onto CuFe_2_O_4_ nanocomposite. (**A**) Langmuir isotherm model; (**B**) Freundlich isotherm plot; (**C**) Temkin isotherm model; (**D**) Dubinin–Radushkevich isotherm plot; and (**E**) equilibrium adsorption capacity against various isotherm models. (Initial pH: 6; initial adsorbate concentration: 40–240 mg/L; dosage of CuFe_2_O_4_ adsorbent: 3.5 g/L; nanocomposite particle size: 742.5 nm; stirring speed: 150 rpm: duration of contact 24 h; operating temperature: 302 K).

An adsorption isotherm is crucial in understanding the interaction of dye anions and nanocomposite particle surfaces at a fixed temperature. An efficient adsorption system must be designed using the adsorption isotherms [47]. The assessment of adsorption isotherms involved the application of four models: the Langmuir, Freundlich, Tempkin, and Dubinin–Radushkevich isotherm models. Table 6 displays the respective model equations and the model parameters. The linearized Langmuir plot (Figure 6A) is better suited to reflect the empirical data compared to other isotherm models (Figure 6B–D), as indicated by the greatest R^2^ value (0.9989) for CR dye uptake onto the CuFe_2_O_4_ nanocomposite adsorbent. Based on the observations from Figure 6A,E, it is evident that the Langmuir isotherm model provides an excellent fit for the experimental CR dye adsorption capacity at equilibrium (q_e_). This suggests that CR dye adsorption follows a uniform monolayer process with a maximum surface adsorption capacity (q_max_) of 64.72 mg/g. Moreover, the obtained separation factor (R_L_) of 0.027–0.076 at all initial adsorbate concentrations (40–240 mg/L) confirms the favorable nature of the adsorption process [48]. The Freundlich constant, n, with a value of 2.535, falls between 1 and 10, further supporting the conclusion that the surface assimilation process is favorable. At elevated concentrations, the process exhibited a greater degree of favorability. In the Freundlich isotherm, the parameters K_f_ and n represent the surface dissimilarity and adsorption intensity, respectively [37]. However, the Freundlich isotherm’s R^2^ was calculated to be 0.9636, indicating that the sorption of CR on the CuFe_2_O_4_ nanocomposite was likely not monolayer adsorption alone but also involved a limited number of chemical interactions. The Temkin isotherm showed strong adsorbent–adsorbate interaction, resulting in an R^2^ value of 0.9858. The positive Temkin constant (R_T_/b_T_) value of 11.42 indicated the endothermic process and the equilibrium binding constant (K_T_ = 3. 417 L/g).

#### Comparison of Several Adsorbents’ Maximal Unimolecular Layer CR Dye Adsorption Capacities as Determined by the Langmuir Isotherm Model

Table 7 compares the maximal unimolecular adsorption capacity of the CuFe_2_O_4_ nanocomposite adsorbent with that of several other adsorbents reported in the literature. Based on the data presented in the table, it can be inferred that the prepared copper ferrite nanocomposite adsorbent exhibits a higher adsorption capacity for the removal of CR from simulated wastewater than that reported for other adsorbents. The increased effective surface area derived from the nanocomposite material is the cause of this better adsorption capacity. According to the findings, CuFe_2_O_4_ is a promising adsorbent for the elimination of color from dye effluents.

### 2.5. Potential Interactions among CuFe_2_O_4_ Nanocomposite Adsorbent and CR Dye Adsorbate

**Figure 7:**
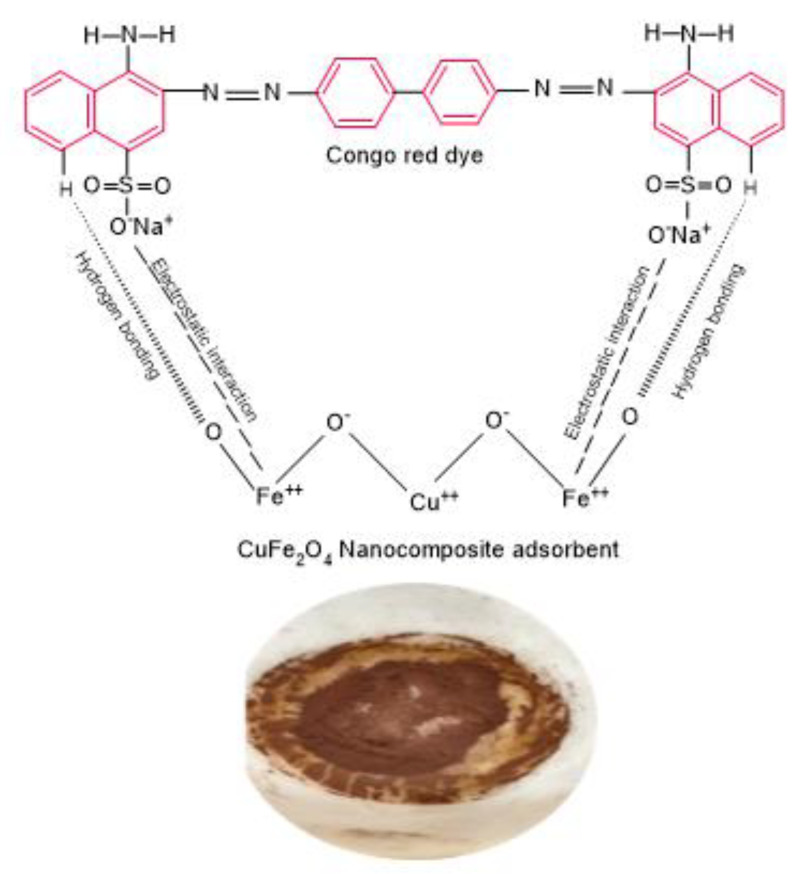
A suggested mechanism for CR dye uptake onto CuFe_2_O_4_ nanocomposite adsorbent.

The interactions between the CR dye and CuFe_2_O_4_ adsorbent play a significant role in an efficient adsorption system; it is crucial to elucidate the adsorption mechanism. The adsorption process is influenced by several fundamental parameters, encompassing the characteristics of functional groups in the nanocomposite adsorbent, the particles’ structural and surface properties, the dye anions’ diffusion behavior toward the particle surface, and the nature of their interaction. The accumulation of dye can take place through either physisorption or chemisorption, depending on the nature of the mutual interaction between the particle surface and the adsorbate. In numerous instances, dye uptake on nanocomposite materials is facilitated by electrostatic interaction, hydrogen bonding, and pore filling [62]. CR is an anionic dye that incorporates a sulfonic group in its structure. This sulfonic group ionizes upon dissolution in an aqueous solution, leading to the creation of colored anions along with aromatic rings. The presence of −SO_3_^−^ anions notably impacts the surface assimilation of CR. The primary interaction mechanism among the positively charged CuFe_2_O_4_ and negatively charged anionic dye molecules (–NH_2_ and –N=N– groups of CR molecule) is proposed to be chemisorption through anion–cation interaction, as shown in Figure 7. Notably, the pH value significantly impacts the dye’s binding ability to the adsorbent [63]. The charge of the CuFe_2_O_4_ nanocomposite may switch from positively charged at a low pH to negatively charged at a high pH, depending on the pH of the initial CR dye solution. Additionally, film diffusion and pore diffusion models have been utilized most frequently to analyze their diffusion mechanisms.

### 2.6. Kinetic Modeling

**Figure 8:**
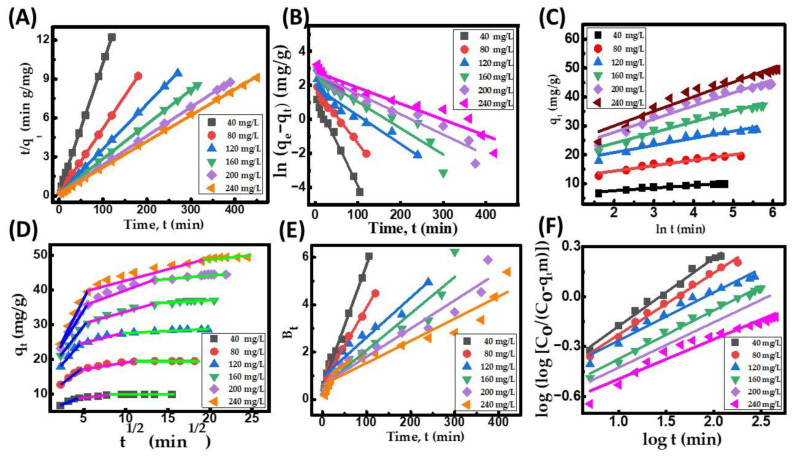
CR dye uptake onto CuFe_2_O_4_ nanocomposite. (**A**) Ho’s pseudo-second-order (PSO) kinetic plot; (**B**) Lagergren pseudo-first-order (PFO) kinetic plot; (**C**) Elovich kinetic plot; (**D**) intraparticle diffusion kinetic model, Blue color (First region) indicates that the external boundary layer diffusion of the adsorbate molecules and the process is rapid, Pink color (Second region) is attributed to the progressive adsorption stage, where pore diffusion is rate-controlling. It indicates the diffusion of the adsorbate molecules through the pores of the adsorbent, Green color (Third region) refers to the final saturation stage and the pore diffusion starts to slow down due to the low adsorbate concentration in the aqueous solution; (**E**) Boyd kinetic plot; and (**F**) Bangham kinetic plot. (Initial pH: 6; initial adsorbate concentration: 40–240 mg/L; dosage of CuFe_2_O_4_ adsorbent: 3.5 g/L; nanocomposite particle size: 742.5 nm; stirring speed: 150 rpm: duration of contact 24 h; operating temperature: 302 K).

Before designing a large-scale adsorption column, it is crucial to conduct an adsorption kinetics study. This study helps to identify the rate-controlling step and process mechanism, which are both significant factors in assessing the overall effectiveness of the process. The kinetics of CR removal onto the CuFe_2_O_4_ nanocomposite adsorbent was investigated by analyzing different kinetic models, including the pseudo-first-order (PFO), pseudo-second-order (PSO), intraparticle diffusion, and Elovich model equations [64]. Table 8 presents the different kinetic parameters of the model used to describe the surface assimilation of the adsorbate onto the nanocomposite particle surface at different initial dye concentrations (40, 80, 120, 160, 200, and 240 mg/L). The kinetics of adsorption for the decolorization of CR dye exhibit two distinct phases: an initial rapid phase followed by a slower final phase. This observation confirms that the active sites of the CuFe_2_O_4_ nanocomposite are easily accessible initially, and, as the process progresses, these sites are gradually and consistently filled at later stages. The quick starting phase could be attributed to the adsorbent’s nanocomposite surface’s occupancy of freely available sites [41]. Equilibrium for the accessible binding sites on the CuFe_2_O_4_ particle surface was achieved at about 120 min for 40 mg/L, 180 min for 80 mg/L, 270 min for 120 mg/L, 315 min for 160 mg/L, 390 min for 200 mg/L, and 450 min for 240 mg/L. Table 8 illustrates that the value of the adsorption capacity at equilibrium and h increased (Figure S3F) with the rise in the initial dye concentration. This can be attributed to the amplified driving force of adsorption among the adsorbate concentration in the feed solution and the adsorbent particle surface resulting from the movement of adsorbate molecules from the feed solution to the adsorbent surface [65]. The increase in the PSO rate constant, K_2_, with a decrease in adsorbate concentration indicates reduced competition for active sites on the surfaces of the adsorbent particles at lower adsorbate concentrations. Figure 8A illustrates the linearized form of the PSO kinetic plot, while the corresponding results obtained from this plot are presented in Table 8. The kinetic plots indicate that the PSO plot (Figure 8A) exhibits higher linearity compared to the PFO (Figure 8B) and Elovich plots (Figure 8C). Based on the results presented in Table 8, it can be inferred that the PSO model is more effective in predicting the removal of dye molecules from the solution. This is supported by the higher coefficient of determination (R^2^) and strong agreement between the computed and predicted q_e_ data for various initial adsorbate concentrations. This suggests that the adsorption process may be governed by chemisorption, where strong binding forces are generated between CR dye molecules and solid particles due to electron distribution [66].

### 2.7. Analysis of CR Dye Uptake Rate Mechanism

The kinetic empirical data are subjected to fitting with the intraparticle diffusion model, and the resulting plot is presented in Figure 8D. The parameters of the model acquired from the fitting process are documented in Table 8. Figure 8D exhibits the plot of q_t_ versus t^1/2^, revealing three distinct linear segments. The initial linear segment adheres to the adsorbate molecules’ external boundary layer movement to the nanocomposite adsorbent particle surface. The rapid initial rate of CR removal can be attributed to the abundance of available sites on the adsorbent surface during the first few minutes of the adsorption process. The CuFe_2_O_4_ adsorbent could adsorb almost 60–65% of CR in 15 minutes, given an initial dye concentration of 240 mg/L. This finding indicates the strong electrostatic interaction between the dye molecules and the outer surface of the solid particles. Additionally, it is suggested that the diffusion rate parameters may exhibit variations with changes in the dye concentration. The second linear segment corresponds to intraparticle diffusion, signifying the diffusion of dye molecules from the nanocomposite adsorbent surface into the pores of the CuFe_2_O_4_ adsorbent particle. This stage represents the progressive adsorption phase, where pore diffusion becomes the rate-limiting step. In the final step, the equilibrium stage is attained when all unoccupied sites in the pores of the solid particle are fully saturated, and the pore diffusion begins to decelerate because of the low dye concentration in the aqueous solutions [67]. Additionally, none of the plots at any given concentration passed through the origin, illustrating that pore diffusion is not the rate-controlling process. The rise in each intercept value with an enhancement in dye concentration suggests that the adsorption process is primarily influenced by external boundary layer movement, with a minor effect on pore diffusion. Thus, the overall rate of the process is predominantly controlled by exterior film diffusion, with little impact on the intraparticle diffusion of CR dye molecules to the solid particle’s interior surface. The investigation revealed that the adhesion process might be governed by external boundary layer movement during the initial stages. As the adsorbent particles accumulate with adsorbate molecules, pore diffusion may dominate the later phases of the process [68]. Furthermore, the collected data underwent analysis based on the kinetic equations proposed by Bangham and Boyd. The Boyd plot (Figure 8E) and Bangham plot (Figure 8F) exhibit non-linear patterns that indicate that the reaction’s total rate is primarily governed by movement in the external film.

### 2.8. Influence of Temperature and Thermodynamic Studies

**Figure 9:**
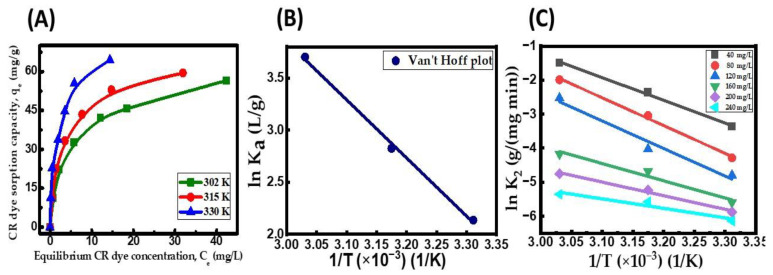
(**A**) Influence of temperature on equilibrium CR dye uptake onto CuFe_2_O_4_ nanocomposite; (**B**) Van’t Hoff plot; and (**C**) Arrhenius plot. (Initial pH: 6; initial dye concentration: 40–240 mg/L; dosage of CuFe_2_O_4_ adsorbent: 3.5 g/L; nanocomposite particle size: 742.5 nm; stirring speed: 150 rpm: duration of contact 24 h; operating temperature: 302–330 K).

Investigating adsorption thermodynamics is crucial in determining the process’ spontaneity and understanding the adsorption phenomenon’s nature. The impact of the temperature on CR adsorption on the CuFe_2_O_4_ nanocomposite was analyzed in the temperature range of 302 to 330 K. Figure 9A displays the results of the equilibrium adsorption amount of dye onto the CuFe_2_O_4_ nanocomposite (q_e_) at different temperatures, along with corresponding saturation dye concentrations (C_e_). The positive correlation between the temperature and the value of q_e_ suggests that the CR dye uptake process is both fast and endothermic [67]. The rise in temperature from 302 to 330 K increased the pore volume of the nanocomposite particle, expanding from 71 mm^3^/g to 79.4 mm^3^/g. Consequently, this allowed a more significant number of adsorbate molecules to penetrate more deeply into the particle’s surface faster. The enhancement in q_e_ at raised temperatures could be attributed to chemical interactions between adsorbate molecules and the CuFe_2_O_4_ nanocomposite or new adhesion active sites on the particle’s surface [69]. The q_max_ of the CuFe_2_O_4_ adsorbent showed an increment from 64.72 mg/g at 302 K to 79.96 mg/g at 330 K. The observed phenomenon can be attributed to the heightened mobility of pollutant molecules across the exterior film and within the interior pores of the solid particle surface. This reduces the swelling of the CR dye molecules as the temperature rises. Additionally, the elevated temperature allows more adsorbate molecules to attain sufficient energy to effectively interact with active sites on the nanocomposite surface [70]. Table 9 provides the thermodynamic parameters determined from the linear fit of lnK_a_ versus the inverse of temperature (Figure 9B). As presented in Table 9, as the temperature rose, the values of ΔG diminished and grew more negative, representing the spontaneity and viability of the adsorption process. Adsorption was proven to be endothermic by the distribution of positive directional values for ΔH_ads_ (46.38 kJ/mole). This was previously indicated by the rise in the q_e_ value with increasing temperature. The Temkin isotherm results were consistent with this finding, showing a positive Temkin constant. The ΔS_ads_ value (0.1726 kJ/(mole K)) being positive indicates a higher degree of arbitrary behavior among adsorbate molecules on the CuFe_2_O_4_ nanocomposite compared to the dye effluent, favoring the interaction between solid and liquid. In the range of initial adsorbate concentrations of 40 to 240 mg/L, the adsorption’s activation energy (Ea) was calculated using the Arrhenius plot (Figure 9C). The computed activation energy values, falling within the range of 42.12 to 67.85 kJ/mole, signify that the binding of adsorbate molecules onto the CuFe_2_O_4_ nanocomposite is a chemisorptive process. This observation may account for the covalent bonds among adsorbate molecules and active sites on the nanocomposite when exposed at raised temperatures [71]. As previously mentioned in Section 2.6, the PSO kinetic model provided further evidence that the chemosorption mechanism was involved in the adsorption process under investigation.

### 2.9. Inference from CuFe_2_O_4_ Nanocomposite Adsorbent Renewal and Reusability Studies

**Figure 10:**
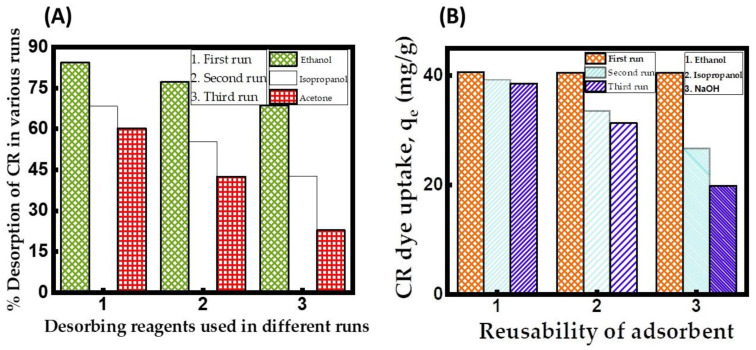
(**A**) Desorption efficacy of CR dye in various runs. (Desorbing reagent volume: 0.1 L; stirring speed: 150 rpm: duration of contact 24 h; operating temperature: 302 K). (**B**) Reusability of CuFe_2_O_4_ adsorbent for the uptake of CR dye in various runs. (Initial pH: 6; initial adsorbate concentration: 175 mg/L; volume of adsorbate solution: 100 mL; nanocomposite particle size: 742.5 nm; stirring speed: 150 rpm: duration of contact 24 h; operating temperature: 302 K).

To evaluate the performance of the regenerated CuFe_2_O_4_, we conducted a study on its dye uptake across three adsorption–desorption processes. The outcomes of the regeneration studies, as depicted in Figure 10A, indicate that the solvent ethanol is a superior desorbing reagent for the regeneration of the CuFe_2_O_4_ adsorbent loaded with CR dye molecules, surpassing other reagents in effectiveness. With different desorbing reagents in consecutive batches, it was found that the order of the percent desorption in all runs was ethanol > isopropanol > acetone. As more runs were performed using ethanol as the solvent, the desorption efficiency of CR revealed a declining trend. The third run yielded the highest desorption efficiency for CR using ethanol, reaching a maximum value of 60.15% (Table S1). A limited solvent volume or inadequate shaking speed might obstruct the disposal of adsorbate molecules from the CuFe_2_O_4_ adsorbent surface into the desorbing reagent. [72]. A reusability study of the adsorption data revealed in Figure 10B demonstrates a progressive decline in q_e_ from 40.62 to 38.57 mg/g when utilizing the CuFe_2_O_4_ nanocomposite recovered with ethanol. Subsequent experiments validate that the regenerated CuFe_2_O_4_ adsorbent maintains dye uptake of up to 38.57 mg/g even after exhaustion, demonstrating its reusability for up to three runs. Upon the third operation, it was discovered that the adsorbent recovered using ethanol maintained 94.95% of its initial adsorption capacity at equilibrium (Table S2). Additionally, when the number of runs increased, the q_e_ value steadily declined. The reasons behind this could be the insufficient removal of the bound adsorbate molecules from the CuFe_2_O_4_ nanocomposite adsorbent and a lack of attachment sites on the solid material [73]. When using ethanol as the solvent, the regenerated CuFe_2_O_4_ nanocomposite adsorbent was effectively reusable for up to three runs, with a gradual decline in adsorption capacity.

## 3. Materials and Methods

### 3.1. Materials Required

The anionic diazo dye CR (dye content ≥35%, purity = 99.98%, and λ_max_ = 498 nm) was procured from Sigma Aldrich, Bengaluru, India. Cupric nitrate trihydrate (Cu(NO_3_)_2_ 3H_2_O, purity = 99.5%), nickel nitrate hexahydrate (Ni(NO_3_)_2_·6H_2_O, purity = 99%), manganese nitrate (Mn(NO_3_)_2_), purity = 99%), ferric nitrate nonahydrate (Fe(NO_3_)_3_·9H_2_O, purity = 98%), and citric acid monohydrate (C_6_H_8_O_7_·H_2_O, purity = 99%) were supplied by Loba Chemie, Mumbai, India. All other chemicals, such as sodium chloride (NaCl, purity = 99.5%), hydrochloric acid (HCl, purity = 99%), sodium hydroxide (NaOH, purity = 99%), ethanol (C_2_H_5_OH, purity ≥ 99%), isopropanol (C_3_H_7_OH, purity ≥ 99.8%), and acetone (CH_3_COCH_3_, purity ≥ 99.9%), were obtained from Merck, Mumbai, India. Every chemical was of analytical reagent quality and applied directly without further purification. All experimental working solutions utilized in the study were prepared using double-distilled water adjusted to the desired pH by adding either 0.1 N HCl or 0.1 N NaOH [35].

### 3.2. Synthesis of Copper Ferrite, Nickel Ferrite, and Manganese Ferrite Nanocomposite Material

The sol–gel method was adopted to synthesize the CuFe_2_O_4_, NiFe_2_O_4_, and MnFe_2_O_4_ nanocomposites. Each metal nitrate (cupric nitrate trihydrate/nickel nitrate hexahydrate/manganese nitrate), ferric nitrate nonahydrate, and citric acid monohydrate was taken in the weight ratio of 1:2:1.5 for the synthesis of each nanocomposite material [28]. The required amounts of nitrates and citric acid were carefully weighed using a digital weighing balance. Each metal nitrate and ferric nitrate nonahydrate was added separately to a glass beaker, and 10 mL of distilled water was added. All the beakers were then placed on a magnetic stirrer, and the solution was stirred at 240 rpm. After the salts were dissolved entirely, citric acid monohydrate was added to all the beakers (as a chelating agent) with roughly 25 mL of distilled water. The solution in each beaker was then heated at 363 K and continuously stirred at 240 rpm until it attained a gel-like consistency. The beaker was then transferred to a hot air oven for sterilization at 363 K (overnight). The obtained dry mixture was transferred to a mortar and pestle. The mixture was ground well and transformed into an excellent powder. The powdered material was then transferred to a crucible and kept inside a muffle furnace for 3 h at 723 K for thermal dissociation and crystal transformation. After 3 h of heating, the crucible was removed from the furnace and cooled naturally [74]. The prepared nanocomposite was transferred to a mortar and pestle, ground to a fine powder, sieved to obtain particles less than 150 µm in size, and stored appropriately in an air-tight plastic container for future studies. Batch experiments were conducted to select appropriate nanocomposite materials to decolorize CR from an aqueous phase.

### 3.3. Analytical Measurements 

The pH of the dye solution was measured by a digital pH meter (Systronics 335, Bengaluru, India), and the average particle size of the copper ferrite nanocomposite adsorbent was evaluated by a nanoparticle size analyzer (Horiba SZ-100, Horiba, Kyoto, Japan). The XPS spectra were recorded on a JEOL JAMP-9500F instrument (JEOL, Tokyo, Japan) equipped with a monochromatic Al Kα X-ray source (15 mA, 14 KV) at 1 × 10^−8^ Torr. The binding energy of the measured spectra was calibrated using the C1s peak at 284.6 eV as a reference peak, and the calibrated spectra were fitted with a mixed Gaussian–Lorentzian function in the XPSPEAK freeware. Magnetometry was performed with a superconducting quantum interference device (Quantum Design, San Diego, CA, USA). Morphological and chemical analyses were recorded on a FE-SEM (JEOL 6300, JEOL, Tokyo, Japan) equipped with EDS elemental mapping. The adsorbent’s surface area and pore volume were determined using a BET surface analyzer (Smart Instruments, Dombivli, India). A pre-calibrated UV/visible spectrophotometer (Shimadzu UV-1800, Shimadzu, Kyoto, Japan) was used to estimate the residual dye concentration in the water sample by measuring the absorbance at 498 nm (λ_max_). FT-IR using a Shimadzu 8400S (Shimadzu, Kyoto, Japan) analyzer in the 400 to 4000 cm^−1^ transmission range was used to determine the functional moieties on the particle surface before and after adsorption. The crystallographic structure of the adsorbent was studied using an XRD pattern analyzer (Rigaku Ultima IV, Tokyo, Japan) in a 2θ range of 0–90°, at a scan rate of 2° min^−1^, with a step size of 0.02°. The thermal stability of the prepared nanocomposite material was measured using TGA (TA Instruments, Newcastle, DE, USA). The salt addition method determined the adsorbent’s pH point of zero charge (pHzpc). In the salt addition method, 0.125 g of CuFe_2_O_4_ nanocomposite was added to 50 mL of 0.05M NaCl solution in an Erlenmeyer flask at different initial pH values from 2 to 12. The mixture was shaken at room temperature (302 K) on a Thermolyne orbital shaker (TOS) (150 rpm) for 24 h. After separating the nanocomposite from the solution, the pH_zpc_ was determined by plotting the Δ_pH_ (initial − final) against the initial pH value, and pH_zpc_ was the initial pH at which Δ_pH_ became 0 (pH at which the nanocomposite passes a net impartial charge) [14,35].

### 3.4. Batch Studies of the CuFe_2_O_4_ Nanocomposites for the Removal of CR Dye

For all decolorization experiments involving the adsorption of CR by the required amount of CuFe_2_O_4_ adsorbent, 250 mL Erlenmeyer flasks with 100 mL CR dye solution were used. Batch adsorption experiments were conducted by altering the level of one experimental parameter while maintaining the constancy of other parameters. A 1000 mg/L stock solution was formulated by dissolving 1 g of CR dye powder in 1000 mL of deionized water. The adsorption process was conducted on a TOS at 150 rpm for varying time intervals at 302 K for 24 h. After adsorption, aliquots from the reaction mixture were subjected to centrifugation (Remi CPR-24 Plus, Mumbai, India) at 12,000 rpm for 10 min, and the remaining adsorbate concentration was determined using a UV/visible spectrophotometer. The measurement involved monitoring changes in absorbance at a wavelength of 498 nm. The equilibrium studies were performed by varying the starting adsorbate concentration with a fixed CuFe_2_O_4_ nanocomposite dosage. The CuFe_2_O_4_ adsorbent capacity for the adsorption of dye uptake at equilibrium (q_e_) was calculated by applying the mass balance equation in Equation (8) [75]:

(8) $${\;q}_{e}=\frac{\left(C_{o}-C_{e}\right)\;V}{W}$$

where C_e_ and C_o_ are the concentrations of the CR dye solution after and before adsorption (mg/L), V refers to the volume of the aqueous phase (L), and W is the mass of the dry nanocomposite adsorbent. The adsorption kinetics were investigated by assessing the residual concentrations of CR dye at 302 K across different contact time intervals. At each interval, 5 mL of the solution was extracted from the Erlenmeyer flask, analyzed for the remaining CR concentration, and promptly returned to the flask to prevent alterations in the volume of the dye solution. All the experiments were repeated in duplicate to verify their reproducibility, and the average results are provided in the Section 2.

## 4. Conclusions

The present research demonstrates the synthesis of a copper ferrite nanocomposite adsorbent and highlights the efficacy, selectivity, and affinity of the adsorbent used to remove toxic Congo red dye effectively from the synthetic aqueous phase. The structural and physical properties of the prepared adsorbent were evaluated based on the results of FT-IR, FESEM/EDS, XRD, TGA, BET, and particle size analysis. Structural and morphological characterizations revealed the CuFe2O4 adsorbent’s porous morphology and spinal cubic structure. The surface morphology was composed of irregular, agglomerate clusters with many small pores. The FT-IR studies revealed that the adsorbent surface possessed the vibration of octahedral group Cu–O stretching, tetrahedral group Fe–O stretching, and bending and stretching vibrations of nitrate. The adsorbent was found to have a point of zero charge (pH_zpc_) of 6.5, indicating that the adsorption of CR was more advantageous in acidic conditions, specifically in the pH range of 5.5 to 6. Several experiments on the batch adsorption process were optimized using a central composite design. The optimum pH, adsorbate concentration, and CuFe_2_O_4_ adsorbent dosage were determined to be 5.5, 125 mg/L, and 3.5 g/L, respectively, for CR dye uptake at equilibrium. The observation revealed that as the temperature and initial adsorbate concentration increased, the equilibrium dye uptake increased, and the value of q_e_ diminished with a rise in particle dosage. The adsorption saturation data fit the Langmuir isotherm model, while the kinetic data fit well with the PSO kinetic model, suggesting a chemisorption mechanism. The most significant match with the computed data in the Langmuir plot for CR dye confirmed the unimolecular layer, which had a q_max_ of 64.72 mg/g at 302 K. The result agrees with those of other reported adsorbents and suggests that higher dye concentrations lead to a more favorable adsorption process. Initially, the dye uptake was governed by external film diffusion, while, at later stages, the rate was predominantly controlled by pore diffusion. The results obtained from the pore diffusion, Bangham, and Boyd kinetic models provide evidence that exterior mobility is the dominant rate-controlling step, effectively governing the overall reaction rate. The measured thermodynamic parameters imply the endothermic and spontaneous nature of the studied adsorbate under adsorption on the CuFe2O4 nanocomposite. The CuFe_2_O_4_ nanocomposite adsorbent can be reused up to three times with ethanol as the solvent, while maintaining dye uptake of 94.95% of its initial adsorption capacity at equilibrium, as confirmed by these regeneration studies. The superior adsorption capacity observed in treating a synthetic dye effluent implies that the copper ferrite nanocomposite adsorbent may hold significant potential for the effective removal of other anionic dyes, heavy metals, drugs, and pesticides from effluents.

## Figures and Tables

**Table 1:** Ranges and levels of different experimental parameters for the adsorption of CR dye onto the nanocomposite.

Experimental Factors	Range and Level		
	−1	0	1
Initial pH (X_1_)	5.5	6.0	6.5
Initial adsorbate concentration, mg/L (X_2_)	125	175	225
CuFe_2_O_4_ nanocomposite dosage, g/L (X_3_)	3.5	4.0	4.5

**Table 2:** Matrix of a central composite design (CCD) with three factors applied to the uptake of CR dye onto CuFe_2_O_4_ nanocomposite. (Nanocomposite particle size: 742.5 nm; stirring speed: 150 rpm; duration of contact: 24 h; operating temperature: 302 K).

Expt. No.	X_1_	X_2_ (mg/L)	X_3_ (g/L)	CR Dye Uptake at Equilibrium, q_e_ (mg/g)	
				Experiment	Predicted
1	0	0	0	40.60	40.70
2	−1	1	−1	40.09	40.76
3	0	1	0	38.00	37.24
4	0	0	−1	45.54	44.60
5	0	0	0	40.49	40.70
6	1	1	1	34.57	34.47
7	0	0	0	40.62	40.70
8	1	−1	1	37.86	37.29
9	−1	0	0	42.18	41.17
10	0	0	0	40.53	40.70
11	−1	−1	1	38.23	38.26
12	−1	−1	−1	48.72	48.92
13	0	0	0	40.59	40.70
14	−1	1	1	33.63	33.74
15	0	0	0	40.56	40.70
16	1	1	−1	39.54	39.60
17	1	−1	−1	46.08	46.07
18	0	−1	0	42.38	42.73
19	0	0	1	36.18	36.71
20	1	0	0	39.49	40.11

**Table 3:** Analysis of variance (ANOVA) for equilibrium uptake of CR dye onto CuFe_2_O_4_ nanocomposite adsorbent based on data derived from central composite design (CCD) experiments conducted within a 2^3^ full factorial design framework.

Term	Coefficient	SE of Coefficient	T_statistics_	DF	Seq SS	Adj SS	Adj MS	F_statistics_	Probability
Constant	40.6965	0.2233	182.276						0.000
Regression				9	246.813	46.813	27.424	65.02	0.000
Linear				3	234.014	34.014	78.005	184.93	0.000
X_1_	−0.5309	0.2054	−2.5850	1	2.819	2.819	2.819	6.68	0.027
X_2_ (mg/L)	−2.7446	0.2054	−13.364	1	75.329	5.329	75.329	178.59	0.000
X_3_ (g/L)	−3.9479	0.2054	−19.223	1	155.866	55.866	155.866	369.53	0.000
Square				3	2.969	2.969	0.990	2.35	0.134
X_1_ × X_1_	−0.0594	0.3916	−0.152	1	0.010	0.010	0.010	0.02	0.882
X_2_ (mg/L) × X_2_ (mg/L)	−0.7069	0.3916	−1.805	1	1.375	1.375	1.375	3.26	0.101
X_3_ (g/L) × X_3_ (g/L)	−0.0416	0.3916	−0.106	1	0.005	0.005	0.005	0.01	0.917
Interaction				3	9.831	9.831	3.277	7.77	0.006
X_1_ × X_2_ (mg/L)	0.4239	0.2296	1.846	1	1.438	1.438	1.438	3.41	0.095
X_1_ × X_3_ (g/L)	0.4701	0.2296	2.047	1	1.768	1.768	1.768	4.19	0.068
X_2_ (mg/L) × X_3_ (g/L)	0.9099	0.2296	3.963	1	6.625	6.625	6.625	15.71	0.003
Residual error				10	4.218	4.218	0.422		
Lack-of-fit				5	4.206	4.206	0.841	355.11	0.000
Pure error				5	0.012	0.012	0.002		
Total				19	251.031				

Regression coefficient, R^2^ = 98.34%, R^2^ (Pred) = 87.04%, R^2^ (adj) = 96.81%, S = 0.6494, PRESS = 32.5366, adequate precision = 17.6433. SE refers to the standard error coefficient; DF is the degree of freedom; Seq SS represents the sequential sum of squares; Adj SS denotes the adjusted sum of squares; Adj MS signifies the adjusted mean squares; PRESS is the predicted residual sum of squares; S is the value on the S chart.

**Table 4:** Verification of the process model for CR dye uptake onto CuFe_2_O_4_ nanocomposite adsorbent. (Nanocomposite particle size: 742.5 nm; stirring speed: 150 rpm; duration of contact: 24 h; operating temperature: 302 K).

Experiment	Process Variables with Operating Conditions			CR Dye Uptake at Equilibrium, q_e_ (mg/g)	
	X_1_	X_2_ (mg/L)	X_3_ (g/L)	Empirical Value	Computed Value
1	6.0	125	4.0	42.34	44.26
2	5.5	125	3.5	48.72	49.38
3	6.5	225	4.5	34.57	35.84

**Table 5:** Optimal values of the experimental factors to achieve the maximum uptake of CR dye at equilibrium. (Nanocomposite particle size: 742.5 nm; stirring speed: 150 rpm; duration of contact: 24 h; operating temperature: 302 K).

Experimental Factors	Optimal Value for CR Dye Uptake	CR Dye Optimum Adsorption Capacity at Equilibrium, q_e_ (mg/g)
Initial pH (X_1_)	5.5	48.72
Initial adsorbate concentration, C_o_ (mg/L) (X_2_)	125	
Dosage of CuFe_2_O_4_ nanocomposite adsorbent, g/L (X_3_)	3.5	

**Table 6:** Equilibrium adsorption isotherm equation and model parameters for the uptake of CR dye onto CuFe_2_O_4_ nanocomposite adsorbent.

Isotherm Model	Linearized Equation	Model Parameters	Values	Model Equation
Freundlich	${\log\;q}_{e}={\;\log\;K}_{F}+\frac{1}{n}\;\log{\;C}_{e}$	n	2.535	$q_{e}=14.799{C_{e}}^{0.3945}$
		K_F_ (L/g)	14.799	
		R^2^	0.95906	
Langmuir	$\frac{1}{q_{e}}=\frac{1}{q_{\max}}+\frac{1}{q_{\max}{\;K}_{L}{\;C}_{e}}$ Separation factor, $R_{L\;}=\frac{1}{1+{\;K}_{L\;}C_{o}\;}$	q_max_ (mg/g)	64.7249	$q_{e}=\frac{19.6038{\;C}_{e}}{1+0.3029{\;C}_{e}}$
		K_L_ (L/mg)	0.3029	
		R^2^	0.9989	
		R_L_	0.027–0.076	
Temkin	$q_{e}=\frac{\mathrm{RT}}{b_{T\;}}{\ln\;K}_{T\;}+\frac{\mathrm{RT}}{b_{T}}\;\ln C_{e}$	RT/b_T_	11.416	$q_{e}=11.416\;\ln\left(3.4175{\;C}_{e}\right)$
		K_T_ (L/g)	3.417	
		R^2^	0.9858	
Dubinin–Radushkevich	$\ln q_{e\;}=\ln q_{s\;}-{\;K}_{\mathrm{DR}}{\;\varepsilon }^{2}\;$ Mean free energy of adsorption, E $E\;=\left[\frac{1}{\sqrt{2K_{DR}}}\right]$	q_s_ (mg/g)	42.125	$q_{e\;}=42.125\;\exp\left(-0.2944\;\epsilon^{2}\right)$
		K_DR_ (mole^2^/kJ^2^)	0.2944	
		E (kJ/mole)	1.303	
		R^2^	0.8317	

**Table 7:** Analysis of highest unimolecular layer surface assimilation capacity (q_max_) of CR dye onto numerous reported adsorbents calculated by the Langmuir isotherm model.

Adsorbent	Highest Surface Assimilation Capacity, q_max_ (mg/g)	Reference
Montmorillonite	12.70	[49]
Fe_3_O_4_@SiO_2_@Zn–TDPAT	17.73	[50]
Fly ash/NiFe_2_O_4_ composite	22.73	[51]
CTAB-modified pumice	27.32	[52]
NiFeTi LDH	29.97	[53]
Chitosan/TiO_2_ nanocomposite	32.00	[54]
Sodium bentonite	35.84	[55]
Coffee husk powder	38.64	[41]
*Cornulaca monacantha* stem biomass	43.42	[56]
Lotus leaf powder	45.89	[57]
*Neurospora crassa* dead biomass with wheat bran	46.29	[58]
NH_2_–Fe_3_O_4_–GO–MnO_2_–NH_2_ nanocomposite	54.95	[59]
Magnetic peanut husk	56.30	[60]
Polycrystalline–Fe_2_O_3_ nanoparticles	58.20	[61]
Copper ferrite nanocomposite	64.72	Present study

**Table 8:** Kinetic equations and model parameters for the uptake of CR dye onto CuFe_2_O_4_ nanocomposite adsorbent.

Kinetic Model	Linearized Equation	Model Parameters	Initial Adsorbate Concentration, C_o_ (mg/L)					
			40	80	120	160	200	240
CR dye adsorption capacity at equilibrium, q_e_, _practical_ (mg/g)			9.819	19.504	28.572	36.980	44.472	49.438
Pseudo-first-order	$\ln\;\left(q_{e\;}–q_{t}\right)={\ln\;q}_{e}-{\;K}_{1}\;t$	q_e_, _computed_ (mg/g)	3.463	6.053	8.712	13.185	14.617	16.474
		K_1_ (1/min)	0.0514	0.0321	0.0167	0.0154	0.0119	0.0094
		Regression coefficient, R^2^	0.9913	0.9877	0.9494	0.9306	0.9474	0.9261
Pseudo-second-order	$\frac{t}{q_{t}}=\frac{1}{K_{2}q_{e}^{2}}+\frac{t}{q_{e}}$ Initial adsorption rate, h = K_2_ q_e_^2^	q_e_, _computed_ (mg/g)	10.0674	19.896	28.346	37.593	45.106	49.925
		K_2_ (g/(mg. min))	0.0344	0.0137	0.0083	0.0037	0.0028	0.0022
		h (mg/(g. min))	3.319	4.228	5.058	5.254	5.549	6.748
		Regression coefficient, R^2^	0.9999	0.9999	0.9999	0.9997	0.9998	0.9996
Intraparticle diffusion	$q_{t}={\;K}_{i}t^{0.5}+∁$	K_i_ (mg/(g. min^1/2^))	0.0879	0.1730	0.1639	0.2307	0.2662	0.3092
		C (mg/g))	8.7274	16.8899	25.6860	32.6852	39.0578	42.4887
		Regression coefficient, R^2^	0.9714	0.8869	0.8898	0.8909	0.9521	0.8967
Elovich	$q_{t}=\left(\frac{1}{\beta }\right)\;\ln\left(\alpha \beta \right)+\left(\frac{1}{\beta }\right)\ln t$	α (mg/(g. min)	263.852	335.522	425.492	516.053	596.372	672.839
		β (g/mg)	1.0772	0.5303	0.4067	0.2712	0.2198	0.1987
		Regression coefficient, R^2^	0.9228	0.9389	0.9138	0.9694	0.9509	0.9465

**Table 9:** Thermodynamic factors for CR dye uptake onto CuFe_2_O_4_ nanocomposite adsorbent.

Temperature (K)	Highest Uptake Capacity of CR onto CuFe_2_O_4_, q_max_ (mg/g)	Thermodynamic Factors		
		ΔG (kJ/mole)	ΔH_ads_ (kJ/mole)	ΔS_ads_ (kJ/(mole K))
302	64.7249	–22.1046	46.3824	0.1726
315	70.7485	–24.2875		
330	79.9634	–26.8990		

where ΔG: standard Gibbs free energy change (=−RT ln K_a_); K_a_ = q_max_ K_L_; ΔH_ads_: changes in enthalpy of adsorption; ΔS_ads_: changes in entropy of adsorption; $\ln K_{a}=\frac{\Delta S_{\mathrm{ads}}}{R}-\frac{\Delta H_{\mathrm{ads}}}{\mathrm{RT}}.$

## Data Availability

The findings of this study can be supported with data that are accessible upon request from the corresponding author.

## Supplementary Materials

The following supporting information can be downloaded at: www.mdpi.com/xxx/s1, Figure S1: Selection of appropriate nanocomposite adsorbent for the uptake of Congo red (CR) dye; Figure S2: Chemical structure of CR dye; Figure S3: CR dye uptake onto CuFe2O4 nanocomposite. (A) Effect of initial pH; (B) effect of shaking speed; (C) influence of adsorbent dosage; (D) influence of particle size; (E) influence of ionic strength; (F) effect of initial adsorbate concentration on CR dye uptake onto CuFe2O4 nanocomposite; Table S1: Regeneration of CR dye-loaded CuFe2O4 nanocomposite adsorbent in various runs; Table S2: Reusability of CuFe2O4 nanocomposite adsorbent for the uptake of CR dye in various runs. References [11,76,77,78,79,80] are cited in the supplementary materials.
