# Supplementary material for: Process Optimization and Equilibrium, Thermodynamic, and Kinetic Modeling of Toxic Congo Red Dye Adsorption from Aqueous Solutions Using a Copper Ferrite Nanocomposite Adsorbent
Source: Molecules. 2024 Jan 15;29(2):0. doi: 10.3390/molecules29020418 (PMC11154345; doi:10.3390/molecules29020418)
Supplement: Supplementary file 1 [file molecules-29-00418-s001.zip › molecules-2793908-supplementary.pdf]

molecules

Supporting Information

Process Optimization and Equilibrium, Thermodynamic, and Kinetic Modeling of Toxic Congo Red Dye Adsorption from Aqueous Solutions Using a Copper Ferrite Nanocomposite Adsorbent

Vairavel Parimelazhagan [1], Akhil Chinta [1], Gaurav Ganesh Shetty [1], Srinivasulu Maddasani [2,*], Wei-Lung Tseng [3,4], Jayashree Ethiraj [5,6], Ganeshraja Ayyakannu Sundaram [7,*] and Alagarsamy Santhana Krishna Kumar [3,8,*]

1 Department of Chemical Engineering, Manipal Institute of Technology, Manipal Academy of Higher Education (MAHE), Manipal 576104, Karnataka State, India; pvairavel@gmail.com (V.P.); akhilchinta14@gmail.com (A.C.); shettygaurav00@gmail.com (G.G.S.)
2 Department of Chemistry, Manipal Institute of Technology, Manipal Academy of Higher Education (MAHE), Manipal 576104, Karnataka State, India
3 Department of Chemistry, National Sun Yat-sen University, No. 70, Lienhai Road, Gushan District, Kaohsiung 80424, Taiwan; tsengwl@mail.nsysu.edu.tw
4 School of Pharmacy, Kaohsiung Medical University, No. 100, Shiquan 1st Road, Sanmin District, Kaohsiung City 80708, Taiwan
5 Department of Physics, School of Arts and Science, AVIT Campus, Vinayaka Mission's Research Foundation, Chennai 603104, Tamil Nadu State, India; dr.jayashreeethiraj@gmail.com
6 CAS in Crystallography and Biophysics, University of Madras, Guindy Campus, Chennai 600025, Tamil Nadu State, India
7 Department of Research Analytics, Saveetha Dental College and Hospitals, Saveetha Institute of Medical and Technical Sciences, Poonamallee High Road, Chennai 600077, Tamil Nadu State, India
8 Faculty of Geology, Geophysics and Environmental Protection, Akademia Gorniczo-Hutnicza (AGH) University of Science and Technology, Al. Mickiewicza 30, 30-059 Krakow, Poland
* Correspondence: s.maddasani@manipal.edu (S.M.); asgchem84@gmail.com (G.A.S.); krishnakumar@mail.nsysu.edu.tw (A.S.K.K.); Tel.: +886-975724627 (A.S.K.K.)

Citation: Parimelazhagan, V.; Chinta, A.; Shetty, G.G.; Maddasani, S.; Tseng, W.-L.; Ethiraj, J.; Ayyakannu Sundaram, G.; Kumar, A.S.K. Process Optimization and Equilibrium, Thermodynamic, and Kinetic Modeling of Toxic Congo Red Dye Adsorption from Aqueous Solutions Using a Copper Ferrite Nanocomposite Adsorbent. *Molecules* **2024**, *29*, x. https://doi.org/10.3390/xxxxx

Academic Editors: Noureddine Barka and Abdelrahman O. Ezzat

Received: 11 December 2023
Revised: 2 January 2024
Accepted: 7 January 2024
Published: date

Copyright: © 2024 by the authors. Submitted for possible open access publication under the terms and conditions of the Creative Commons Attribution (CC BY) license (https://creativecommons.org/licenses/by/4.0/).

2. Materials and Methods

Regeneration and Reusability Studies

An adsorbent is regarded as effective and economical if it exhibits significant retention of adsorbate removal efficiency and adsorption capacity after multiple runs. The quantity of utilized adsorbent must be reduced to keep costs to a minimum and the process can be completed through regenerating the adsorbent material from the treated effluent. Thus, desorption studies were executed to evaluate the efficiency of desorbed adsorbate from the adsorbent particle surface and the recovery of exhausted adsorbent. In this study, we explored the process of desorption and regeneration of CR-laden $CuFe_2O_4$ nanocomposite using various desorbing reagents, such as ethanol, isopropanol, and acetone to regenerate the adsorbent [1]. The loaded CR dye molecules on $CuFe_2O_4$ nanocomposite adsorbent were exposed to the various desorbing reagents in separate batches to achieve the desired outcome. To make sure the adsorbent for the decolorization experiment was fully saturated, the optimum amount of adsorbent dosage was placed in a 250 mL Erlenmeyer flask with 100 mL of 200 mg/L initial concentration of CR dye solution. The adsorbate solution with solid adsorbent is then shaken at 150 rpm for 24 h. We examined the removal efficiencies of the regenerated $CuFe_2O_4$ nanocomposite adsorbent during three cycles of adsorption-desorption processes to assess its performance. After reaching equilibrium, the used adsorbent is centrifuged and dried at 338 K in each flask before being subjected to various desorbing reagent (100 mL) treatments in separate batches and being reused to test its reusability for subsequent runs [2].

3. Results and Discussion

3.1. Selection of Appropriate Nanocomposite Material for the Removal of CR Dye from Simulated Effluent

Batch adsorption experiments were carried out using various nanocomposite materials such as copper ferrite, nicker ferrite, and manganese ferrite at room temperature to evaluate the adsorption capacity and the results are revealed in Figure S1.

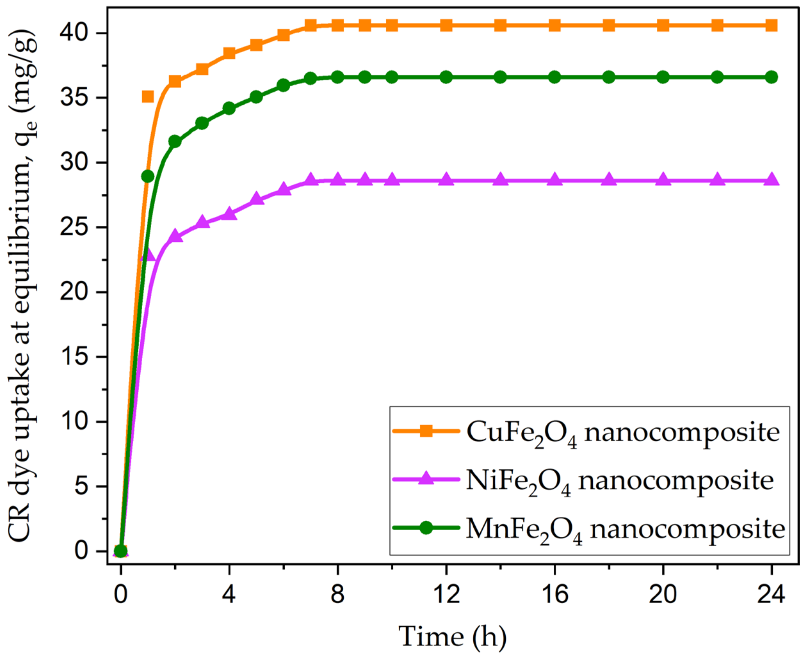

Figure S1. Selection of appropriate nanocomposite adsorbent for the uptake of CR dye. (Initial pH: 6; initial adsorbate concentration: 200 mg/L; dosage of $CuFe_2O_4$ adsorbent: 4 g/L; nanocomposite particle size: < 1000 nm; stirring speed: 150 rpm: duration of contact 24 h; operating temperature: 302 K).

Figure S1 shows that the maximum equilibrium dye uptake of CR was observed using the adsorbent copper ferrite with an initial adsorbate concentration of 200 mg/L at pH 6 and 24 h. This might be because the copper ferrite adsorbent particle has more active sites available. In comparison to other nanocomposite materials, the copper ferrite has a greater surface area and pore volume at 30.03 m^2/g and 71 mm^3/g, respectively. Therefore, among three different adsorbent choices, copper ferrite nanocomposite was discovered to have higher outcomes and was investigated for further studies.

3.2. Analysis of Batch Adsorption Experiments for the Removal of CR Dye from Synthetic Wastewater

3.2.1. Effect of Initial pH of Dye Solution on Adsorption Behavior

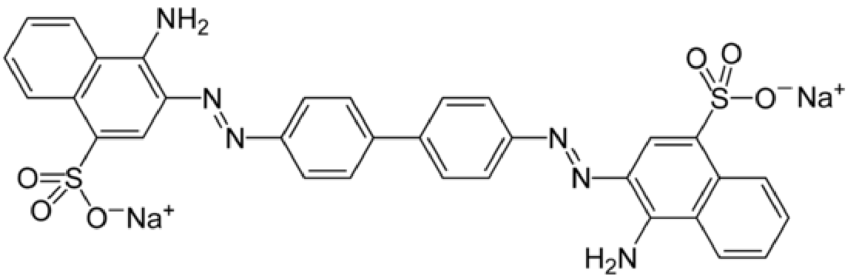

Figure S2. Chemical structure of Congo red (CR) dye.

3.2.2. Effect of Agitation Speed

The shaking speed in a batch adsorption process plays a crucial role in overcoming external boundary layer resistance. To understand its impact on dye uptake, the shaking speed was varied from 0 to 180 rpm at room temperature. From Figure S3B, it was observed that as the agitation speed increased, the equilibrium adsorption capacity of CR showed a significant improvement, rising from 15.83 mg/g to 40.84 mg/g. A decrease in the thickness of the film resistance around the particles of the $CuFe_2O_4$ nanocomposite adsorbent may have augmented turbulence, which increased external film diffusion and CR dye molecules uptake, resulting in the rise in adsorption capacity. This occurrence can be explained by the larger contact surface area created between the adsorbent and the adsorbate solution due to the increased agitation speed. As a consequence, more dye molecules are diffused from the aqueous solution to the vacancy sites of the adsorbent, leading to the higher adsorption capacity observed in the experiment [5].

3.2.3. Effect of $CuFe_2O_4$ Nanocomposite Adsorbent Dosage

The equilibration of the adsorbent and adsorbate is significantly influenced by the nanocomposite dosage. Figure S3C shows that the CR dye uptake at equilibrium decreases from 77.61 mg/g to 28.60 mg/g with increasing the adsorbent dose from 1 to 6 g/L. This is mainly due to the concentration gradient between the adsorbate in the bulk solution and the adsorbate in the liquid phase at equilibrium. In addition, the adsorption of dye molecules on the particle surface, no change in the surface area of the adsorbent particle, and the competition for the availability of binding sites for the accumulation of dye decreases with the rise in the nanocomposite adsorbent dosage. This phenomenon could be caused by adsorbent particle interactions, such as aggregation or overlapping of adsorption binding sites, which are brought on by high adsorbent concentration. The overall active surface area of the adsorbent would be reduced as a result of such aggregation, and the length of the diffusion path would be increased [6].

3.2.4. Effect of $CuFe_2O_4$ Nanocomposite Adsorbent Particle Size

Figure S3D shows the impact of adsorbent particle size on equilibrium dye uptake and it was observed that the CR dye adsorption capacity gradually increased from 39.78 mg/g to 41.13 mg/g as the particle size diminished from 1074.8 to 465.2 nm. The gradual increase in dye uptake may be attributable to smaller particles' higher surface areas per unit mass that are available for adsorption. Additionally, the reduced size of the particles contributes to a shorter diffusion path, enabling the dye molecules to penetrate more deeply into the adsorbent particles at a rapid rate. As a result, an increase in dye uptake is achieved due to the enhanced accessibility and interaction between the dye and the smaller adsorbent particles [7].

3.2.5. Effect of Electrolytes

An adequate number of dissolved inorganic salts such as sodium chloride, sodium bicarbonate, and sodium nitrate are present in the effluent discharge from the textile industry, which may have an impact on the uptake of adsorbate molecules. As a result, it's crucial to research how ionic strength affects the adsorption process in wastewater treatment in textile industries. Various concentrations of the above-mentioned inorganic salts were introduced in separate batches to examine the impact of ionic strength on the equilibrium dye uptake of CR. Figure S3E depicts that the CR dye uptake at equilibrium improved with a rise in the concentration of ionic strength. This is explained by the fact that there is now more positive charge on the particle surface of the nanocomposite adsorbent, which decreases the electrostatic repulsion between the molecules of CR dye and the adsorbent. The rise in ionic strength leads to a greater aggregation of adsorbate molecules, and in turn, encourages the adsorption of CR [8].

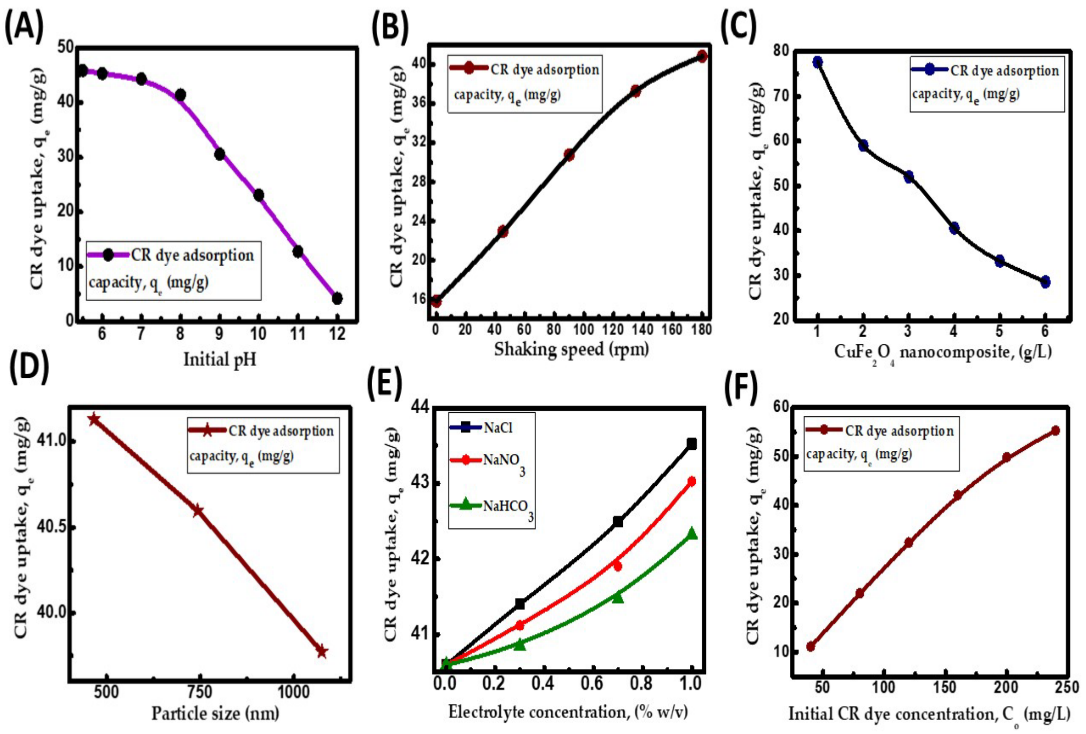

Figure S3. CR dye uptake onto $CuFe_2O_4$ nanocomposite (A) Effect of initial pH; (B) Effect of shaking speed; (C) Influence of adsorbent dosage; (D) Influence of particle size; (E) Influence of ionic strength; (F) Effect of initial adsorbate concentration on CR dye uptake onto $CuFe_2O_4$ nanocomposite.

3.3. Inference From $CuFe_2O_4$ Nanocomposite Adsorbent Renewal and Reusability Studies

Table S1. Regeneration of CR dye-loaded $CuFe_2O_4$ nanocomposite adsorbent in various runs.

Sl. No	Desorbing reagent used to regenerate $CuFe_2O_4$ nanocomposite	Desorption efficiency (%) of CR from $CuFe_2O_4$ loaded adsorbent		
1	Ethanol	84.341	68.340	60.149
2	Isopropanol	77.229	55.279	42.559
3	Acetone	68.554	42.650	22.840

Table S2. Reusability of $CuFe_2O_4$ nanocomposite adsorbent for the uptake of CR dye in various runs.

Sl. No	Desorbing reagent used to regenerate $CuFe_2O_4$ nanocomposite	CR dye uptake onto regenerated $CuFe_2O_4$ adsorbent at equilibrium, q_e (mg/g)		
1	Ethanol	40.617	39.230	38.567
2	Isopropanol	40.587	33.574	31.332
3	Acetone	40.597	26.720	19.857

References

1. Patel, H. Elution profile of cationic and anionic adsorbate from exhausted adsorbent using solvent desorption. *Sci. Rep*. **2022**, *12*, 1665. https://doi.org/10.1038/s41598-022-05805-8
2. Rose, P.K.; Kumar, R.; Kumar, R.; Kumar, M.; Sharma, P. Congo red dye adsorption onto cationic amino-modified walnut shell: Characterization, RSM optimization, isotherms, kinetics, and mechanism studies. *Groundw. Sustain. Dev.* **2023**, *21*, 100931. https://doi.org/10.1016/j.gsd.2023.100931
3. Alsoaud, M.A.; Taher, M.A.; Hamed, A.M.; Elnouby, M.S.; Omer, A.M. Reusable kaolin impregnated aminated chitosan composite beads for efficient removal of Congo red dye: isotherms, kinetics and thermodynamics studies. *Sci. Rep*. **2022**, *12*, 12972. https://doi.org/10.1038/s41598-022-17305-w

4. Shahat, A.; Kubra, K.T.; El-marghany, A. Equilibrium, thermodynamic and kinetic modeling of triclosan adsorption on mesoporous carbon nanosphere: Optimization using Box-Behnken design. *J. Mol. Liq.* **2023**, *383*, 122166. https://doi.org/10.1016/j.molliq.2023.122166
5. Benjelloun, M.; Miyah, Y.; Bouslamti, R.; Nahali, L.; Mejbar, F.; Lairini, S. The fast-efficient adsorption process of the toxic dye onto shells powders of walnut and peanut: Experiments, equilibrium, thermodynamic, and regeneration studies. *Chem. Afr.* **2022**, *5*, 375–393. https://doi.org/10.1007/s42250-022-00328-1
6. Salihi, S.A.; Jasim, A.M.; Fidalgo, M.M.; Xing, Y. Removal of Congo red dyes from aqueous solutions by porous γ-alumina nanoshells. *Chemosphere* **2022**, *286*, 131769. https://doi.org/10.1016/j.chemosphere.2021.131769
